# Sensory Stimulation of the Foot and Ankle Early Post-stroke: A Pilot and Feasibility Study

**DOI:** 10.3389/fneur.2021.675106

**Published:** 2021-07-05

**Authors:** Alison M. Aries, Valerie M. Pomeroy, Julius Sim, Susan Read, Susan M. Hunter

**Affiliations:** ^1^School of Allied Health Professions, Faculty of Medicine and Health Sciences, Keele University, Keele, United Kingdom; ^2^Acquired Brain Injury Recovery Alliance (ABIRA), School of Health Sciences, University of East Anglia, Norwich, United Kingdom; ^3^National Institute for Health Research (NIHR) Brain Injury MedTech Co-operative, Cambridge, United Kingdom; ^4^School of Nursing and Midwifery, Faculty of Medicine and Health Sciences, Keele University, Keele, United Kingdom

**Keywords:** stroke, rehabilitation, somatosensory stimulation, lower extremity, motor recovery, feasibility study, sensory retraining

## Abstract

**Background:** Somatosensory stimulation of the lower extremity could improve motor recovery and walking post-stroke. This pilot study investigated the feasibility of a subsequent randomized controlled trial (RCT) to determine whether task-specific gait training is more effective following either (a) intensive hands-on somatosensory stimulation or (b) wearing textured insoles.

**Objectives:** Determine recruitment and attrition rates, adherence to intervention, acceptability and viability of interventions and outcome measures, and estimate variance of outcome data to inform sample size for a subsequent RCT.

**Methods:** Design: randomized, single-blinded, mixed-methods pilot study.

**Setting:** In-patient rehabilitation ward and community.

**Participants:** *n* = 34, 18+years, 42–112 days following anterior or posterior circulation stroke, able to follow simple commands, able to walk independently pre-stroke, and providing informed consent.

**Intervention:** Twenty 30-min sessions of task-specific gait training (TSGT) (delivered over 6 weeks) in addition to either: (a) 30–60 min mobilization and tactile stimulation (MTS); or (b) unlimited textured insole (TI) wearing.

**Outcomes:** Ankle range of movement (electrogoniometer), touch-pressure sensory thresholds (Semmes Weinstein Monofilaments), motor impairment (Lower Extremity Motricity Index), walking ability and speed (Functional Ambulation Category, 5-m walk test, pressure insoles) and function (modified Rivermead Mobility Index), measured before randomization, post-intervention, and 1-month thereafter (follow-up). Adherence to allocated intervention and actual dose delivered (fidelity) were documented in case report forms and daily diaries. Focus groups further explored acceptability of interventions and study experience.

**Analysis:** Recruitment, attrition, and dose adherence rates were calculated as percentages of possible totals. Thematic analysis of daily diaries and focus group data was undertaken. Standard deviations of outcome measures were calculated and used to inform a sample size calculation.

**Results:** Recruitment, attrition, and adherence rates were 48.57, 5.88, and 96.88%, respectively. Focus groups, daily-diaries and case report forms indicated acceptability of interventions and outcome measures to participants. The 5-m walk was selected as primary outcome measure for a future trial [mean (SD) at end of intervention: 16.86 (11.24) MTS group and 21.56 (13.57) TI group]; sample size calculation indicated 60 participants are required per group.

**Conclusion:** Recruitment, attrition and adherence rates and acceptability of interventions and outcomes justify a subsequent powered RCT of MTS+TSGT compared with TI+TSGT.

## Introduction

Every 2 s, someone in the world experiences a stroke; there are more than 1.2 million stroke survivors in the United Kingdom (UK) alone (1). Many stroke survivors—between 65% (2) and 85% (3)—experience somatosensory impairment. This impacts adversely on the ability to detect, discriminate, and recognise sensations from the body because somatosensory function includes tactile sensation, vibration, pressure, proprioception, temperature, and pain (4). Somatosensory impairment of the lower limb is experienced by between 45% (5) and 56% (6) of stroke survivors and makes performance of everyday tasks difficult (5, 7). Consequently, potential for achieving independent walking post-stroke is decreased (8).

Regaining the ability to walk is a priority for many stroke survivors. Identifying best treatments to address balance, gait, and mobility has been identified by the James Lind Alliance as one of the top 10 research priorities for stroke (9). Progress is promised by interventions aiming to reduce motor impairment and thus recovery of body functions toward their pre-stroke state by utilising the principles of activity-driven neuroplasticity (10). Interventions to facilitate activity-driven neuroplasticity are placed into a framework of priming, augmentation, and practise (11).

Priming interventions prepare the sensorimotor system for motor function, specifically when limited or no volitional control of movement exists. Priming can be achieved through the provision of somatosensory stimulation as a precursor to task-specific training (11). Therapists can deliver intensive proprioceptive and tactile stimulation through a hands-on intervention known as mobilization and tactile stimulation (MTS) (12). Research into MTS for the contralesional hand post-stroke found reduction of motor impairment and improved upper-limb function (13, 14). MTS is also applied to the foot (15, 16). It is hypothesised that greater somatosensory awareness and alignment of the foot, through intensive somatosensory stimulation using MTS, improves the ability to place and transfer weight over the foot, permitting adaptation to different floor surfaces. However, this has not yet been tested.

Augmenting interventions may also enhance somatosensation during task-specific activity. For example, standing on textured materials (17) and wearing textured insoles (TIs) in shoes to improve perceptual motor performance (18). TIs are designed to stimulate sensory receptors on the plantar surface of the foot: tactile (19), pressure (20), and vibration (21). Afferent information from the foot and ankle is, therefore, crucial for postural control and walking capacity (22). TIs that enhance sensory awareness of the foot during motor activity are also expected to improve contact of the foot with the supporting surface and thus interaction between the foot and the floor, which is important for functional activity (23). The use of TIs is a “hands-off,” low-cost augmentation strategy that has been shown to reduce mediolateral sway in healthy populations (24), and change spatiotemporal gait parameters in people with multiple sclerosis (25). However, TIs have not yet been investigated in a stroke population, stimulating the contralesional side.

Practice interventions use task-specific training, which is recommended when stroke survivors can repeat and practice movements or tasks (11). Task-specific training has been shown to improve motor function post-stroke (26–28). More specifically, task-specific gait training (TSGT) is an effective intervention after stroke (26, 29–31).

It is known that afferent input can influence motor control (32, 33). However, it is not known whether combining TSGT with somatosensory stimulation—either priming using MTS, or augmentation by wearing TIs—would increase the effect. The hypothesis is that MTS (priming intervention) immediately before TSGT has greater efficacy than TSGT combined with wearing TIs (augmentation intervention) in reducing sensorimotor impairment and improving functional ability of the more paretic lower limb after stroke. Before this hypothesis can be tested in an adequately powered randomized controlled trial (RCT) it was important to undertake a pilot study to determine the viability of a subsequent RCT (34, 35).

The objectives for this study were to:

1. Estimate recruitment rate for a subsequent RCT.
2. Estimate attrition rate for a subsequent RCT.
3. Estimate the adherence rate to the interventions and their acceptability to participants.
4. Investigate acceptability and feasibility (effective delivery and success of blinding) of a battery of outcome measures, to inform primary and secondary outcome measures for a future trial.
5. Undertake a sample size calculation for a subsequent RCT, using the estimated variance of the selected primary outcome measure.
6. Monitor the type and frequency of adverse events.

## Materials and Methods

### Design, Randomization, Ethics, Trial Registration, and Procedure

We undertook a two-group, randomized, single-blinded pilot study with an embedded qualitative investigation using participant self-report in daily diaries, and focus groups.

Randomization of participants to one of two groups (MTS+TSGT or TIs+TSGT) was undertaken by the Norwich Clinical Trials Unit, using a computer randomization system in a 1:1 ratio with permuted blocks of two and four. Stratification by left- or right-sided brain lesion (identified from the medical notes) was used because this factor may influence rehabilitation potential (36). Group allocation order was concealed from the research team until after a participant had completed the measurement battery at baseline (37).

All outcome measurements in which observer bias could occur were undertaken by assessors blinded to treatment group allocation. Measures were employed to enhance blinding. To avoid a chance observation of insoles unblinding the assessor, all participants were provided with one pair of insoles, but those in the MTS+TSGT group were told not to use them. On completion of the interventions all the TIs were collected and observed for any indications that they had been worn. In addition, participants were asked not to disclose group allocation to the blinded assessor and the case report forms were not accessible to the blinded assessor.

Research ethics approval for this study was obtained from the UK National Research Ethics Service (4/3/16), IRAS No: 171968/REC Ref 16/WM/0080. All participants gave informed consent. The study was registered on a clinical trials database (ISRCTN 13676183; Central Portfolio Management System ID 30449). The study sponsor was Keele University.

**Figure 1:**
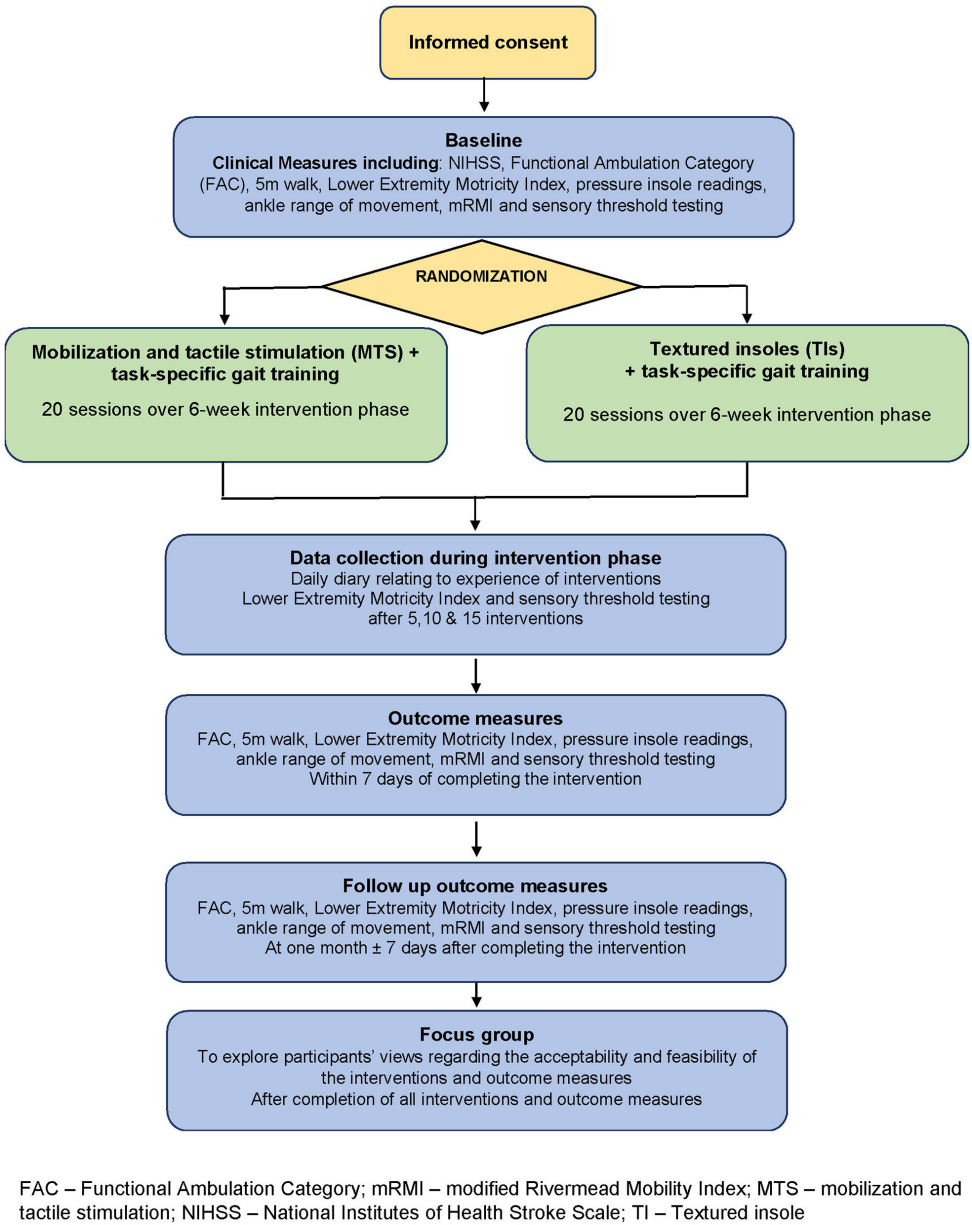
Overview of the study design. FAC, Functional Ambulation Category; mRMI, modified Rivermead Mobility Index; MTS, Mobilization and tactile stimulation; NIHSS, National Institutes of Health Stroke Scale; TI, Textured insole.

Following eligibility screening and after providing written informed consent, participants undertook the measurement battery at baseline (prior to randomization), after 20 sessions of intervention (end of intervention), and 1 month thereafter (follow-up). Progress measures within the intervention phase were also recorded after 5, 10, and 15 treatments, and participants kept a daily diary during the intervention phase. Post-study focus groups explored participant acceptability and feasibility of the interventions and outcome measures. An overview of the study is given in Figure 1.

### Participants

We recruited adults (aged 18 years or older) with stroke occurring in any brain area 42–112 days prior to providing informed consent. Specific inclusion criteria were: able to follow simple commands and imitate actions using the less paretic (ipsilesional) upper limb; able to walk independently prior to index stroke with or without a walking aid; and unable to step on and off a 7·5 cm high block more than 12 times in 15 s with either the more paretic (contralesional) or less paretic (ipsilesional) lower limb (38). Exclusion criteria were: any pathology or previous stroke affecting lower-limb sensation e.g., diabetic neuropathy; fixed contracture of the tendo Achillis; pressure sores or ulcers on the foot or contralesional ankle; any pathology affecting blood circulation to or from the contralesional foot; botulinum toxin injection to the contralesional lower limb in the previous 6 months; pain sufficient to prevent undertaking interventions or outcomes; and known HIV, hepatitis non-A or related condition (to meet sponsor requirements).

Where posterior circulation stroke had caused paresis in either the ipsilesional side or both sides of the body, prior to randomization we consulted the participant's therapist, whose opinion was accepted in relation to identifying the more paretic side.

### Sample Size, Setting, and Recruitment

A sample of 34 was considered achievable and adequate to enable a sample size calculation for the subsequent RCT by estimating variance on a continuous outcome measure (39, 40), accounting for 10% attrition (objective 5).

The study was conducted in an in-patient stroke rehabilitation ward and community-based Stroke Early Supported Discharge service. Potential participants were identified by research nurses or multidisciplinary stroke teams. Stroke survivors (42–112 days post-stroke) expressing an interest in participating were given a participant information sheet. The first 34 stroke survivors meeting inclusion criteria and providing written informed consent were recruited. The study interventions and outcome measurements were performed either in an inpatient clinical setting within an NHS organisation or at the participant's own home, depending upon where the participant resided at that time. However, wherever possible the location for undertaking the outcome measures at baseline was replicated for the end of intervention and 1-month measures.

### Interventions

Each group received one of two interventions, either MTS+TSGT or TIs+TSGT: 20 sessions delivered within a 6-week period. If participants were receiving routine therapy (usual care), this continued alongside the study interventions. Dose and content of routine therapy were recorded on a treatment record (41) by the clinical team.

Standardisation of the three components of the two interventions was facilitated by developing protocols, co-produced with experienced therapists, using a modified nominal group technique (42), and the Template for Intervention Description and Replication (TIDieR) checklist (43). This was a “dynamic iterative process, involving stakeholders, and reviewing published research evidence, drawing on existing theories” [(44) p. 1].

Research therapists (*n* = 4) received training in applying and delivering all interventions. To assess fidelity to protocol, the research therapists were observed by a senior member of the project team (SMH) at various points during the study to ensure they were working to protocol.

### Mobilization and Tactile Stimulation for the More Paretic Lower Limb

**Figure 2:**
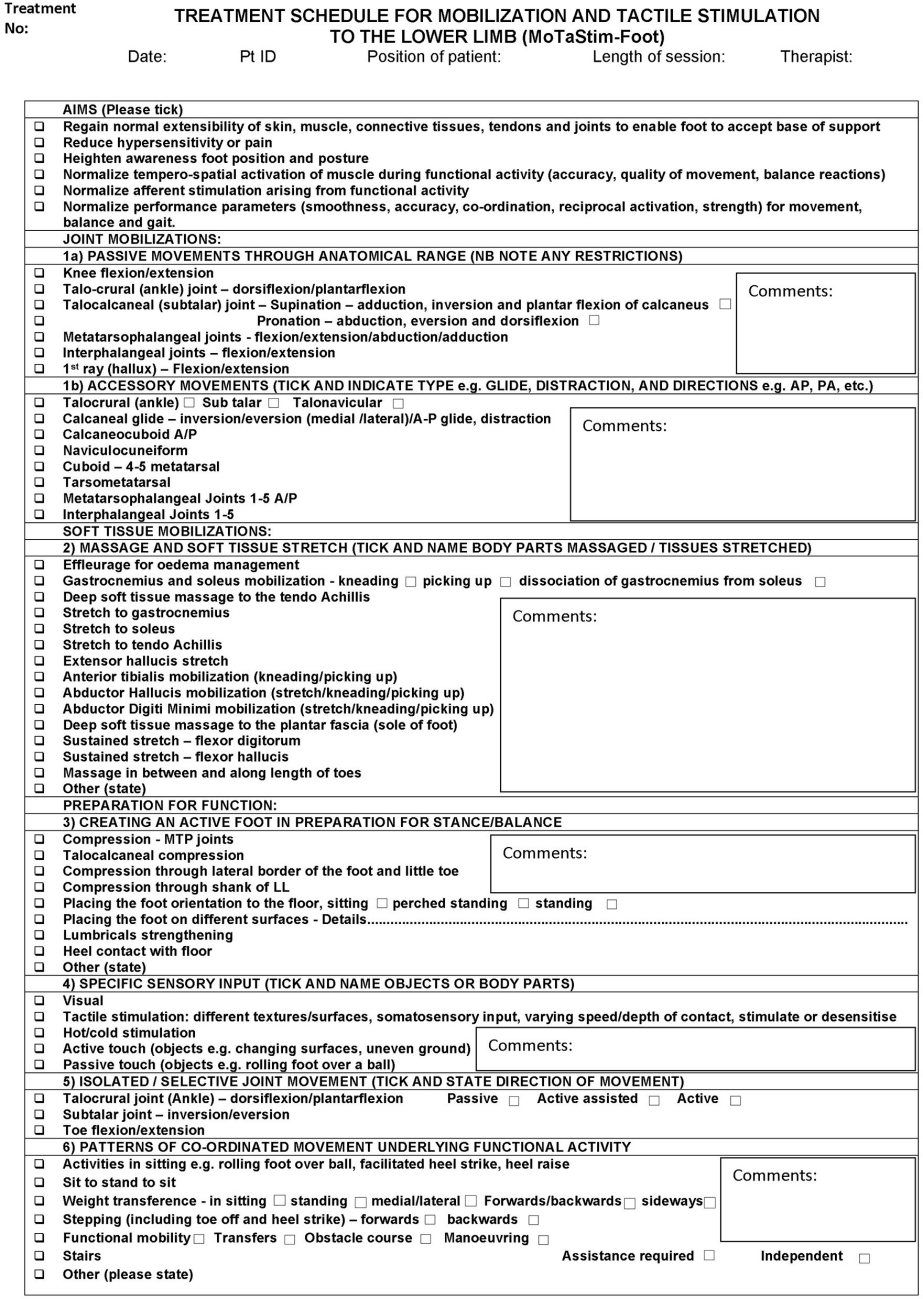
Treatment schedule for mobilization and tactile stimulation to the lower limb.

MTS+TSGT group participants received 30–60 min of standardised lower-limb MTS [based upon a dose-response study of MTS for the upper limb (45)] (Figure 2) immediately prior to TSGT. MTS consisted of physical therapy techniques from six intervention categories: (1) passive and accessory movements of joints, (2) massage and soft tissue stretch, (3) creating an active foot in preparation for stance/balance, (4) specific sensory input, (5) isolated/selective joint movement, and (6) patterns of coordinated movement underlying functional activity. The MTS schedule is a module rather than a single intervention, with flexibility to provide individualised interventions to each participant within the confines of the module. Research therapists selected and delivered appropriate combinations of techniques to address specific clinical needs of participants, influenced by multiple issues (46), thereby reflecting clinical reality. Selections and combinations were based upon clinical decisions; it was neither necessary nor appropriate to deliver all techniques in the schedule to all participants. This allowed consideration of issues such as foot hypersensitivity and tolerance of techniques.

### Wearing a Textured Insole in the Shoe of the Contralesional Lower Limb

**Figure 3:**
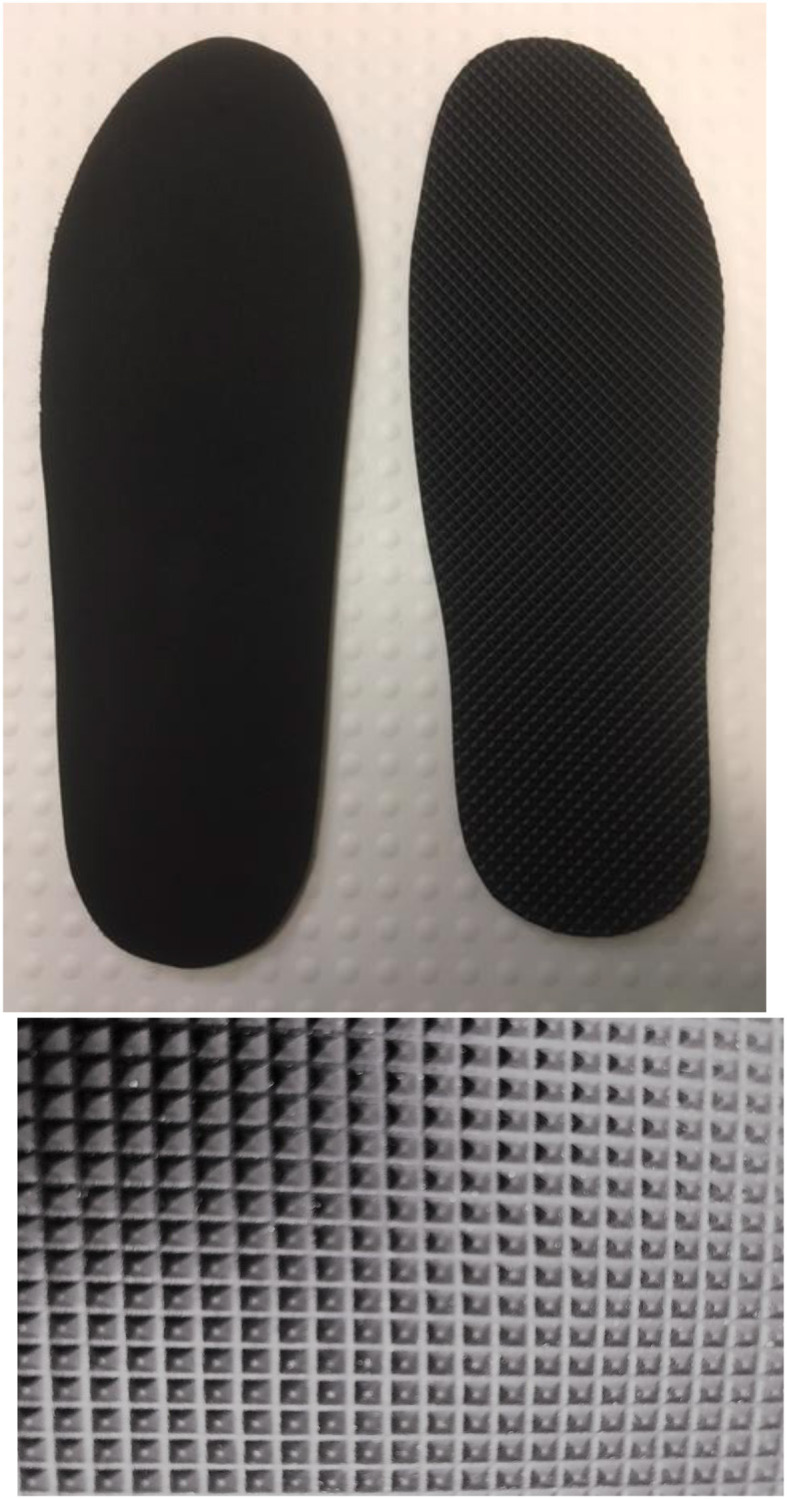
Smooth and textured insole (TI), with a close-up of the pyramidal peaks of the textured material.

For TI+TSGT group participants, one TI was placed in the shoe worn on the contralesional foot, and one smooth insole in the shoe worn on the ipsilesional foot. The TI was black, OG1549, with small, pyramidal peaks, with centre-to-centre distances of ~2.5 mm Evalite Pyramid EVA, of 3-mm thickness, Shore value A50 (Figure 3). The smooth insole consisted of medium density EVA, 3-mm thickness, Shore value A50, black, OG1304. Both insoles were made from material manufactured by Algeos UK Ltd., Liverpool, UK^1^.

Insoles were participant-specific and cut to size to fit in each individual shoe. The TI protocol is presented in Table 1. In summary, participants were encouraged to wear the insoles daily for as long as they chose, with advice to gradually increase wearing duration during the 6-week intervention period. They were provided with daily diary sheets to note wearing time (MTS and TI daily diaries in Supplementary Material).

**Table 1:** Protocol for the textured insole group (based on the Template for Intervention Description and Replication, TIDieR checklist).

1	Name:	Textured insole (TI) protocol for TI group
2	Rationale:	The plantar (sole of the foot) mechanoreceptors are key, sending information to the CNS; plantar stimulation has been shown to result in increased control of body sway (47). In view of the importance of cutaneous information from the plantar surface of the foot to control balance (48), other potential mechanisms of increasing plantar stimulation have been explored. TIs improve postural control in standing in healthy participants (49), walking patterns for people with multiple sclerosis (25). However, the combination of wearing TIs and task-specific gait training (TSGT) has not been evaluated to determine the benefits for balance and walking recovery early after stroke. The use of TIs in the shoes of stroke survivors involves a hands-off (therapist independent) approach, which may potentially be a more economical option for achieving increased sensory stimulation to the foot and is, therefore, important to investigate.
3	Materials:	Smooth and TIs will be patient specific and cut to size so they fit in the participant's shoe. Both insoles are manufactured by Algeos UK Ltd., Liverpool, UK. The smooth surface will be of medium density EVA, 3 mm thickness, Shore value A50, black, OG1304. The TI has small, pyramidal peaks with centre-to-centre distances of ~2.5 mm Evalite Pyramid EVA, 3 mm thickness, Shore value A50, black, OG1549.
4	Procedures:	This group of participants will be encouraged to wear the TI on the contralesional side (and a smooth insole the opposite side), as much as possible (to “augment” the sensorimotor system), during the 4–6-week intervention period, except when the outcomes are being assessed. In addition to wearing the TIs participants will also receive 20 sessions of TSGT (30 min each session), during the intervention period. The specific content of each treatment session will be documented; daily diaries will inform the research team of the extent of wearing of the TIs. Outcome measures will be undertaken without the participant wearing TIs, so it is the same conditions as the mobilization and tactile stimulation group, which is the other arm of the trial.
5	Provided by:	Participant is responsible for wearing textured/smooth insoles. If help is needed to put TIs into shoes and put on footwear (and no family support is available), a Research Therapist will assist, prior to TSGT.
6	Mode of delivery:	Participant controlled—wearing the TIs for as much as possible during the 4–6-week intervention period. The Research Therapist will help if required to put the TIs into shoes and put on footwear prior to TSGT if required. The participant will, therefore, wear the TIs a minimum of 30 min 4–5 times per week.
7	Location:	The participants will be encouraged to wear the TIs and receive the TSGT in their own environment, whether this is as an inpatient or their own home.
8	When and how much:	Time wearing the insoles will vary. Some participants may just wear them during the TSGT and others may wear them for long periods in the day. Participants will be encouraged to record the length of time insoles are worn on the daily diaries.
9	Tailoring:	The participant is in control of time wearing insoles, tailoring intervention to their comfort and needs.
10	Modifications:	Any modifications to the TI protocol will be recorded.
11	Intervention adherence and fidelity:	**Planning:** Intervention adherence and fidelity will be assessed. Strategies to improve fidelity and adherence include: research therapist training, encouragement and motivation regarding wearing of TIs by the research therapist delivering the TSGT and the keeping of daily diaries which will be collected weekly. A record of the length of time wearing the insoles will be included as a prompt in the simple diaries and this information will enable monitoring of adherence and fidelity. The information from the daily diaries will be analysed by the Chief Investigator, with additional guidance from the research team.
12	Intervention adherence and fidelity:	**How well, actual:** The analysis of the daily diary sheets will give an indication of adherence to the intervention. The focus groups will enable further opportunity of assessing the adherence, fidelity and acceptability of the intervention.

*TI, Textured insole; TSGT, Task-specific gait training*.

### Task-Specific Gait Training

Thirty minutes of TSGT were delivered to all participants in both groups, either immediately following MTS treatment (MTS+TSGT group), or while participants wore the insoles (TI+TSGT group), in accordance with the TSGT protocol (Table 2). The specific content of each gait-training session was documented using the comprehensive list of TSGT interventions described (TSGT record in Supplementary Material).

**Table 2:** TSGT protocol based on the Template for Intervention Description and Replication (TIDieR) checklist.

1	Name:	Task-specific gait training (TSGT) group.
2	Rationale:	Walking is a priority for many stroke survivors, confirmed by studies undertaken to define a national research agenda, which identified physical therapy to address balance and gait (walking) post-stroke within the top 10 research priorities [James Lind Alliance, (9)]. There is strong evidence that task-specific walking practice can be used to improve walking after stroke (50).
3	Materials:	Based upon a review of the literature and a focus group with experienced clinicians a few pieces of equipment will be required including theraband, football, chair, foam cushion, gym ball a stair or step and a wobble board.
4	Procedures:	30 min of TSGT will be supervised by the research therapist, with 20 sessions being delivered over a 4–6-week intervention period. The TSGT will be undertaken immediately after the mobilization and tactile stimulation (MTS) for the MTS group. The TSGT will be undertaken whilst wearing a TI on the contralesional side and a smooth insole on the other side, for the TI group
5	Provided by:	The TSGT will be delivered by a research therapist (Band 6), with experience of working with stroke patients. A log will be kept of which research therapist provides which treatment for each participant and this information will be analysed on completion of the trial.
6	Mode of delivery:	The research therapist will provide the TSGT in a 1:1 situation.
7	Location:	The TSGT will take place in either an inpatient clinical setting within an NHS organisation or a University research setting or the participant's own home.
8	When and how much:	All participants in both groups/arms of the trial will receive 20 sessions of 30 min of TSGT within a 4–6-week period.
9	Tailoring:	Although a standardised protocol will be followed for the TSGT the research therapist will choose appropriate exercises and adapt them as required to suit the requirements of each individual participant, due to differences in presentation following a stroke. This reflects how TSGT would usually be implemented in conventional rehabilitation. Details of actual intervention delivered will be recorded on the treatment schedule.
10	Modifications:	Any modifications to the TSGT protocol will be monitored and reported appropriately.
11	Intervention adherence and fidelity- planned:	Intervention adherence and fidelity will be analysed. Strategies to improve fidelity and adherence include 1:1 intervention plus encouragement and motivation strategies by the research therapist during the TSGT, as in usual therapy rehabilitation. A log will be kept detailing, for each participant, which research therapist has delivered the TSGT.
12	Intervention adherence and fidelity—how well (actual):	The case report files completed by the research therapists will give an indication of adherence to the intervention. The focus groups will enable further opportunity of assessing the adherence, fidelity, and acceptability of the intervention.

*MTS, Mobilization and tactile stimulation; TSGT, Task-specific gait training*.

### Data Collection

Originally, we intended to undertake blood flow studies using a portable ultrasound machine to explore whether changes occur in response to MTS treatment. However, this proved not to be feasible due to sonographer time constraints.

### Participant Characteristics at Baseline

Data recorded at baseline were: participant demographics; clinical characteristics, including the National Institutes of Health Stroke Scale (NIHSS) (51)^2^; self-report of walking distance; and the Functional Ambulation Category (52).

### Intervention Fidelity and Adherence to Protocol

Delivery of MTS was recorded according to length of each session (minutes). The number of sessions delivered was also recorded. Adherence to protocol was accepted if participants received at least 30 min of MTS (and <60 min) in each intervention session. Understanding optimal treatment dose, as advocated by the second Stroke Recovery and Rehabilitation Roundtable (53), is important to inform future trials.

Participants recorded duration of wearing insoles each day in their daily diary, and it was also reported in case report forms. Delivery of TSGT was recorded according to length of each session (minutes) and adherence calculated as the number of sessions within 10% of the 30 min specified in the protocol.

### Lower-Limb Sensorimotor Impairment and Functional Ability

Table 3 provides full details of measures of lower-limb sensorimotor impairment and functional ability and time points for their administration.

**Table 3:** Summary of outcomes for the MoTaStim-Foot pilot study.

**Outcome**		**Measurement tool**	**Frequency of measurement**	**Additional information**
Sensorimotor impairment	Ankle range of motion—dorsiflexion/plantarflexion and inversion/eversion	Electrogoniometer attached to lower leg (lateral border) contralesional side	Baseline Post-intervention 1-month follow-up	Provides ratio-level data (cm) Intra-rater reliability r = 0.979 (54).
	Touch/pressure sensory threshold of plantar skin—under heel, hallux, 1st metatarsal phalangeal (MTP) joint and 5th MTP joint	Semmes Weinstein monofilaments (SWMs), using a bespoke algorithm (protocol in Supplementary Material)	Baseline After 5, 10, and 15 treatments, Post-intervention 1-month follow-up	Provides ordinal data; filaments are numbered 1–20. One represents the largest force (300 g, 6.65) and 20 the smallest force (0.008 g, 1.65). Intra-rater reliability has been reported to be an r value of >0.9 when a specific protocol was followed (55).
	Motor impairment (strength) of hip flexors, knee extensors and ankle dorsiflexors	Lower Extremity Motricity Index (LEMI)	Baseline After 5, 10, and 15 treatments, Post-intervention 1-month follow-up	Provides interval level data. For individual actions (ankle dorsiflexion, knee extension and hip flexion) and all actions combined, Pearson correlations—good to excellent (r = 0.78–0.91), significant (*p* < 0.001) (56). Excellent test-retest intra-rater reliability of the Lower Extremity Motricity Index (LEMI) as a measure of strength (ICC = 0.93) (57).
Lower-limb function and balance	Walking ability	Functional Ambulation Category (FAC)	Baseline Post-intervention 1-month follow-up	Provides categorical/nominal data. Valid and responsive, with excellent intra-rater reliability (Cohen *k =* 0.950) and inter-rater reliability (*k =* 0.905) in stroke survivors (52).
	Walking speed	Modified 5-metre walk test (5MWT) (videoed)	Baseline Post-intervention 1-month follow-up	Provides ratio-level data (seconds). 5MWT was shown to have a standardised response mean (95%CI) of 1.22 (0.93, 1.50) at a comfortable pace and 1.00 (0.68, 1.30) at a maximum walking pace (58)
	Pressure under the feet during stance phase of walking	TEKSCAN™ (F-Scan™) pressure insoles to record force-time integral (FTI) and centre of force velocity (COFV) in an AP direction	Baseline Post-intervention 1-month follow-up	Provides ratio-level data force time integral (FTI) (N/sec) and (COFV) (cm/sec). Foot Scan pressure insole systems have been found generally to provide reliable force and pressure data (ICC > 0.75) (59). Pressure insoles (the Parotec System) have shown a very strong degree of association with an Advanced Mechanical Technology Inc. (AMTI) force plate when measuring COP in an AP direction with Pearson's correlation coefficients—greater than 0.90 for 67/67 trials (100%) (60).
	Functional mobility	Modified Rivermead Mobility Index (mRMI)	Baseline Post-intervention 1-month follow-up	Provides ordinal level data. Inter-rater reliability excellent = 0.98 (*p < *0.001) (61). The minimal clinically relevant difference is 4.5 points.
Participants' perceptions of the acceptability of the interventions and outcome measures		Daily diaries and focus groups	Information recorded daily throughout the intervention period. Attendance at a focus group on completion of all the interventions and measures	Focus groups were used to provide an insight into the participants' trial experiences (62). Topic guides were used. A patient and public involvement and engagement (PPIE) advisor assisted with note-taking and summarising the information discussed at the end of each focus group. Braun and Clarke's (63) six-stage process for thematic analysis was broadly followed.

*5MWT, Five metre walk test; AP, Anterior-posterior; Cm, Centimetres; COFV, Centre of force velocity; COP, Centre of pressure; FAC, Functional Ambulation Category; FTI, Force time integral; ICC, Intraclass correlation co-efficient; LEMI, Lower Extremity Motricity Index; N/sec, Newtons per second; SWMs, Semmes Weinstein monofilaments*.

To meet objective 4 and enable selection of a primary outcome measure and sample size calculation (objective 5), a battery of outcome measures was chosen. In relation to the International Classification of Functioning, Disability and Health (ICF) (64), the interventions delivered aimed to alter body structures and functions and activity; participation was also explored through the focus groups. We considered previously reported appropriateness of each outcome measure for measuring sensorimotor impairment or lower-limb function and balance, as well as attention to validity, reliability and responsiveness to change (65, 66).

### Sensorimotor Impairment

Clinically relevant measures of sensorimotor impairment were selected to explore sensory or motor changes based upon potential mechanisms of effect, to enable effectiveness to be explored in a future study. Wherever possible, procedures were standardised, and staff were trained appropriately.

In view of the potential for TIs to augment somatosensation and facilitate sensorimotor control whilst being worn, we removed these from footwear before undertaking outcome measure assessment to ensure parity of testing conditions for both groups.

The Lower Extremity Motricity Index (LEMI), a valid and reliable (56, 57) measure with no cost implications—with scores ranging from 0 (no movement) to 33 (full strength) for each of three joints tested in a limb, i.e., hip, knee and ankle; a final one point is added so it can be scored out of 100 (57)—was used according to a standardised protocol developed for the trial to monitor strength of hip and knee flexors and ankle dorsiflexors.

We measured ankle range of movement during gait and analysed ankle dorsiflexion and inversion during stance phase, using an electrogoniometer^3^, costing £4,611, attached to the lateral border of the lower leg. The sensor was positioned vertically in alignment with the lateral malleolus; the data log acquisition unit was calibrated (zeroed) with the participant standing in a neutral position (67). Maximum range of ankle dorsiflexion and inversion movement was recorded during the 5-metre walk test (5MWT); range of knee motion was not measured. Data were extracted from the Biometrics programme and run through a Matlab programme to provide results in degrees of movement. The participants were videoed during the 5MWT to give a visual representation of gait for interest; the video was available for participants to view if they wished to.

Touch/pressure sensory thresholds in the feet relate to activity outcomes (68) and Semmes Weinstein monofilaments (SWMs), costing £240, were therefore selected as an outcome measure for this study. SWMs are a reproducible method of assessing somatosensation (55). Touch/pressure sensory threshold was established by identifying the minimum force required to accurately identify touch/pressure threshold and was measured using SWMs at four anatomical points located on the plantar surface of the foot: mid-heel; pad of the hallux; 1st metatarsophalangeal (MTP) joint; and 5th MTP joint (56). We developed a bespoke protocol informed by the literature to reduce potential testing fatigue and this was used successfully following training of assessors (Supplementary Material).

Pressure under the feet was measured during stance phase of walking using the TEKSCAN™ (F-Scan™) system, costing ~£14,000, which involved inserting thin insoles (containing 960 separate pressure-sensing cells arranged in rows and columns) into participants' shoes. Following calibration, force-time integral and centre of force velocity (COFV) were measured (59). Symmetry of gait was not a focus of this pilot study.

### Functional Ability

We selected clinically relevant measures of lower-limb functional ability to explore changes in function, specifically balance and walking, which had potential to enable effectiveness to be explored in a future study.

The Functional Ambulation Category (FAC), which had no cost implications, measured walking ability (scored from 0, non-functional ambulator to 5, ambulator, independent) (52); the research assessor selected the appropriate category after observing the participant walk. The 5MWT, which also had no cost implications, measured walking speed (58). However, the environment for undertaking assessment and standardisation of the measure should be considered. Suzuki et al. (69) advocated a 3-metre (m) lead-up (thus a total of 8 m), and Salbach et al. (58) advised 2 m to allow acceleration, and a further 2 m at the end for deceleration (thus a total of 9 m). This straight distance is not usually available in participants' homes; a modified 5MWT was, therefore, conducted.

Functional mobility (bed mobility, transfers, sitting and standing balance, walking and stairs) was assessed using the modified Rivermead Mobility Index (mRMI), which again had no cost implications (61). A maximum score of 40 is possible for the mRMI. Owing to lack of access for stair assessments in some homes, to maintain parity across participants, we omitted the stairs section when calculating the mRMI (maximum score 35). The full validated mRMI measure was, therefore, not used.

### Observer Blinding

At 1-month follow-up, the blinded assessor was asked to indicate to which group she thought the participant had been allocated (reported as a percentage of correctly guessed group allocations); this allowed us to assess the success of observer-blinding procedures.

### Daily Diaries

Throughout the intervention period, participants were asked to keep a daily diary to help them to “focus their thoughts” [(70), p. 993] and record any changing perception of their lower limb (sensation, movement, or function), and their experiences of interventions and outcomes. These qualitative data aimed to gain an understanding of acceptability of the interventions and outcome measures (objectives 3 and 4).

### Focus Groups

After follow-up data had been collected, all participants were invited to attend one of two focus groups, according to group allocation (two focus groups for each intervention group), exploring their study experiences (62) and the acceptability of the intervention and outcome measures (objectives 3 and 4). A focus group topic guide, developed by the research team and patient and public involvement and engagement (PPIE) advisors, included topics relating to: participation in the study; acceptability of interventions and outcome measures; and perceived changes in lower-limb functions of standing, balancing, and walking following intervention. The topic guide was influenced by an understanding of changes that may occur when somatosensory stimulation is delivered. Open questions facilitated greater understanding of participants' experiences, enabling them to take a lead in sharing information (71). Separate topic guides were developed for each group.

MTS+TSGT group (Supplementary Material);

TI+TSGT group (Supplementary Material).

Following consent, focus groups were audio-taped, and moderated by one researcher (AMA). A PPIE advisor, trained to undertake the role, took field notes. At the end of the discussion, the moderator summarised the key issues, with assistance from the PPIE advisor, and sought validation from participants (62). Debrief meetings with PPIE volunteers and members of the research team immediately after each focus group enabled discussion of the main topic areas, identification of preliminary themes, and topics (71) that needed further exploration in the second focus group for that group. Debriefing also involved evaluation of the moderator's role and consideration of researcher reflexivity (71). Consideration and bracketing of the lead researcher's assumptions (from extensive clinical experience prior to undertaking the focus groups) continued throughout data collection and analysis phases (72). Debriefing meetings offered the opportunity for psychological support for PPIE volunteers in case of unpredictable emotional response (73) to issues raised during the focus group.

### Adverse Events and Reactions

Adverse reactions of pain and fatigue were monitored, informing about acceptability and appropriateness of interventions for stroke survivors in future studies (objective 6). Overly intensive TSGT has potential to elicit pain or fatigue in the contralesional lower limb post-stroke. Therefore, pain and fatigue were monitored and documented on the case report forms throughout the study, using verbal report for pain and decrease in LEMI score for fatigue.

### Analysis

As this was a pilot study, no between-group analysis or formal hypothesis testing were undertaken (74, 75).

### Objectives 1 and 2

We calculated recruitment and attrition rates from the number of patients invited to participate, the number and proportion of those consenting to participate, the number and proportion of eligible participants recruited, and the number and proportion of those lost to follow-up at 1-month, which was the longest feasible follow-up time for the study.

### Objective 3

We assessed adherence to intervention protocols (MTS, wearing TIs, and TSGT). The mean amount of TSGT delivered per group was calculated, as well as mean duration of MTS and time wearing TIs. Adherence was confirmed if the interventions had been delivered to protocol.

### Objective 4

Participant acceptability, including comfort and ease of use, of each outcome measure was assessed using data from daily diaries and focus groups. We assessed effective delivery and appropriateness of primary and secondary outcome measures in terms of feasibility of administering, ability to standardise procedures, clinical relevance, cost, psychometric properties and level of measurement, presence of floor/ceiling effects, responsiveness to change, as well as the number and proportion of participants completing the outcome measures and variance of scores of the measures (65).

Feasibility and acceptability of delivering interventions and outcome measures were informed by calculating frequency of pain, fatigue, and adverse events, and from participant feedback (daily diaries and focus groups). The target was that outcome measures would be completed for all participants at all three time points and be acceptable to the participants. The results from both quantitative and qualitative data were analysed and synthesised to inform final decisions.

We undertook content analysis (76) of daily diary data and thematic analysis for both focus group and daily diary data, using Braun and Clarke's (63) six-stage process, to explore acceptability of interventions (MTS, TIs, and TSGT) and outcome measures (objectives 3 and 4). Once initial coding had taken place, data management was facilitated using NVivo qualitative data analysis (Software QSR International Pty Ltd. Version 11, 2016). Organisation and familiarisation of data and codes was also enhanced by using the one-sheet-of-paper method (77).

One researcher (AMA) coded the transcribed data and subsequently discussed codes, themes and sub-themes with two other members of the research team (SMH and SR), who had also coded transcripts independently, identifying provisional themes for discussion. These provisional themes were shared and discussed amongst the research team and an independent PPIE representative (who had read all focus group transcripts), enabling maturation of themes. Any differences of opinions were discussed. The research team collectively looked for conceptual relationships within and across the focus groups. Final themes were identified from both the daily diaries and focus groups. We kept an audit trail of how themes were developed and matured.

The number and proportion of participants completing all outcome measures were calculated; floor and ceiling effects were also explored from the data.

### Objective 5

Variance of outcome measure scores was calculated, providing information for a sample size calculation for a future main trial.

### Objective 6

Adverse events and reactions were recorded.

### Summarising the Results of the Study

A traffic light system (78, 79) indicating what aspects of the study should be taken forward and any that need modification was developed. Aspects that should not be retained were graded red, those that could be retained but with some changes, amber and those that could be retained without any changes green.

### Study and Data Management

A Study Management Group monitored the study for adherence to protocol and timelines. Quality assurance was supported by the Norwich Clinical Trials Unit. We collected and stored all data securely in accordance with the General Data Protection Regulation 2018 (80).

## Results

### Participant Characteristics

A total of 34 participants were recruited to the trial. Participant characteristics are presented in Table 4. No differences were discernible between the MTS+TSGT and TI+TSGT groups at baseline.

**Table 4:** Participant characteristics and demographics at baseline.

		**MTS (*n* = 19)**	**Textured Insole (*n* = 15)**	**All (*n* = 34)**
Age (years)	Mean (SD)	73.8 (14.1)	72.4 (9.8)	73.2 (12.2)
Sex	Male; *n* (%)	9 (47.4)	9 (60.0)	18 (52.9)
	Female; *n* (%)	10 (52.6)	6 (40.0)	16 (47.1)
Type of stroke	Ischemic; *n* (%)	17 (89.5)	12 (80.0)	29 (85.3)
	Haemorrhagic; *n* (%)	2 (10.5)	3 (20.0)	5 (14.7)
Side of brain lesion	Left (%)	11 (57.9)	9 (60.0)	20 (58.8)
	Right (%)	8 (42.1)	6 (40.0)	14 (41.2)
Days after stroke	Mean (SD)	59.5 (18.1)	53.9 (12.4)	57.0
	Range	43–106	43–95	43–106
Walking prior to stroke	% able to walk >1 mile prior to stroke	68.4	73.3	70.6
NIHSS	Median (IQR)	6.00 (4.00, 7.25)	5.00 (4.00, 7.00)	5.00 (4.00, 7.00)
	Range	1–11	3–16	1–16
FAC	Median (IQR)	4 (2, 5)	3 (1, 4)	3 (2, 4.25)
	Range	1–4	1–4	1–4

*FAC, Functional Ambulation Category; MTS, Mobilization and tactile stimulation; NIHSS, National Institutes of Health Stroke Scale; TI, Textured insole*.

### Recruitment Rate, Flow Through Study, and Attrition Rate (Objectives 1 and 2)

**Figure 4:**
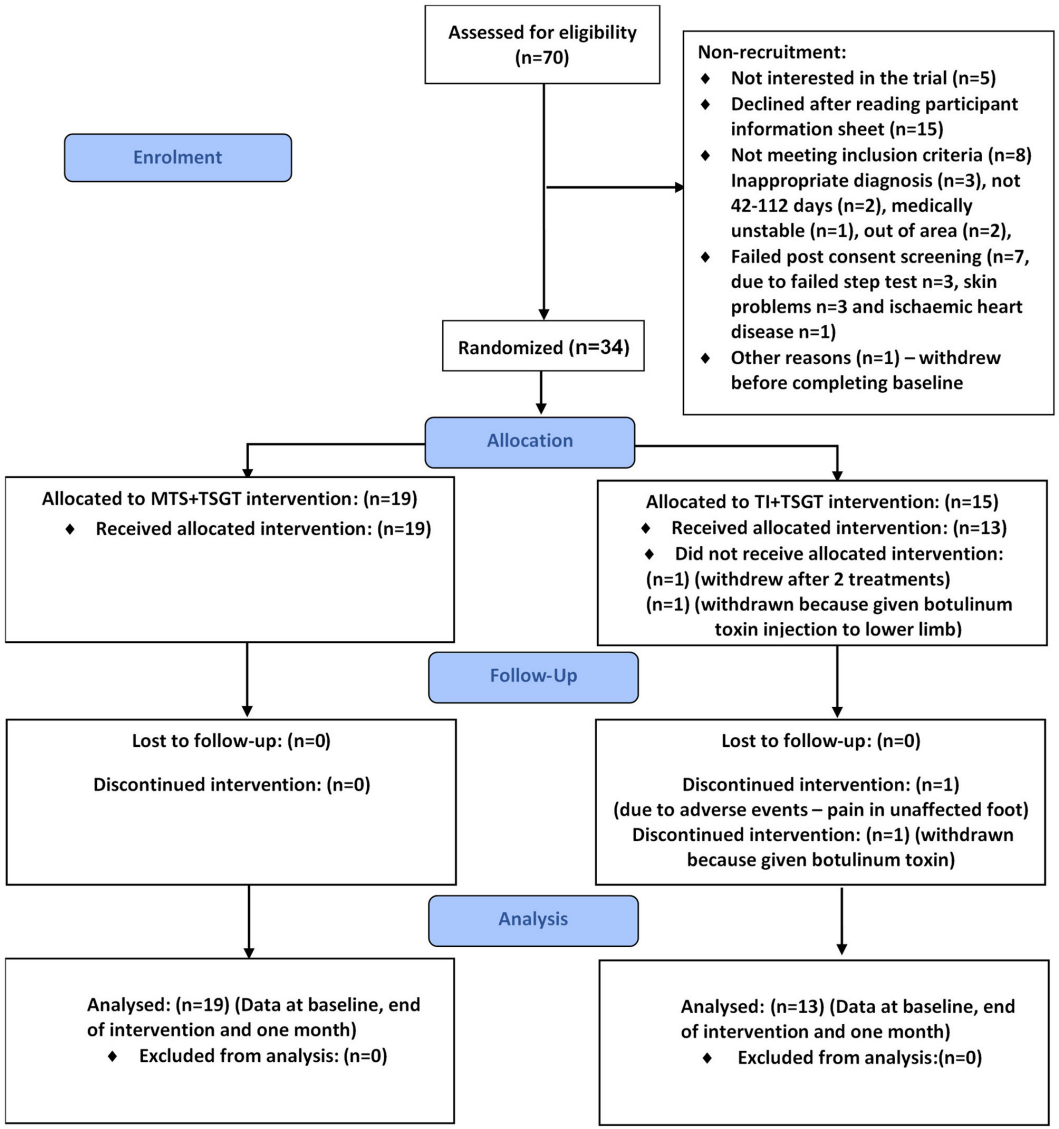
CONSORT diagram for the MoTaStim-Foot feasibility study.

Over the 18-month recruitment period, we assessed 70 stroke survivors for eligibility. Of these, 34 (48.57%, 95% CI 37.2%, 60.0%) were recruited into the study and randomized to one of the two interventions: 19 participants to receive MTS+TSGT and 15 to receive TI+TSGT (Figure 4).

Attrition rate was 5.88% (95% CI 1.6%, 19.1%) at both outcome and follow-up timepoints. Two participants were withdrawn from the TI+TSGT group: one received a botulinum toxin injection to the contralesional lower limb prior to completing the first treatment session, and another developed a lesion (unrelated to the trial) on the ipsilesional foot. Consequently, 32 participants completed outcome and follow-up measures.

### Interventions Adherence Rates and Acceptability (Objective 3)

All participants, apart from two withdrawn from the TI+TSGT group, received all 20 sessions. All research therapists were observed to be delivering the interventions to protocol.

The dose of each intervention and usual care delivered, including actual dose achieved and adherence rate for interventions, and the amount of routine therapy (usual care) delivered can be seen in Table 5. The mean (SD) number of minutes of routine therapy delivered per participant was similar for both groups [MTS+TSGT group, 44.44 (12.48); TI+TSGT group, 43.28 (14.61)].

**Table 5:** Dose of each intervention and usual care delivered, including actual dose achieved (fidelity) and adherence rate for interventions.

	**MTS+TSGT**	**TI+TSGT**	**USUAL CARE**
Number (and range) of sessions	All participants (*n* = 19) received 20 sessions of both MTS and TSGT	13 participants received all 20 sessions; one participant received two TSGT sessions (2–20)	MTS+TSGT: 181 (0–22) TI+TSGT: 195 (0–43)
Mean (SD) number of sessions per participant	20.00 (0.00)	18.62 (4.99)	MTS+TSGT: 9.53 (5.62) TI+TSGT: 15.00 (11.99)
Prescribed dose (minutes)	MTS: 30–60 TSGT: 30	TIs: >30 TSGT: 30	N/A
Actual dose delivered (fidelity) [mean (SD); range in minutes]	MTS: 31.15 (3.47); 22–54 TSGT: 29.03 (2.58); 18.75–30.20	TIs: 462 (48); 30–720 TSGT: 30.21 (0.92); 29.25–32.65	MTS+TSGT: 44.44 (12.48) [Lower limb focused 37.71 (21.31); upper limb focused 19.85 (22.62)] TI+TSGT: 43.28 (14.61) [lower limb focused 44.86 (15.65); upper limb focused 16.98 (22.39)]
Adherence rate^*^	MTS: 94.74% TSGT: 94.74% (18 of 19 participants)	TIs: 100% TSGT: 100% (all 13 participants)	N/A

*Measured as percentage of intervention delivered to protocol i.e., 30–60 min of MTS; >30 min of wearing TI*.

* *TSGT—within 10% of the specified 30 min; N/A, not applicable*.

*MTS, Mobilization and tactile stimulation; SD, Standard deviation; TI, Textured insoles; TSGT, Task-specific gait training*.

### Adherence and Fidelity

Prescribed dose, actual dose delivered (fidelity), and adherence rates to protocol for delivery of MTS, TI, and TSGT are explicit in Table 5. Adherence rate for all the interventions was good, ranging from 94.74% (MTS) to 100% (TIs).

The lowest value of time for wearing the TIs was 0.5 h; this occurred because one participant was unable to “don and doff” the TIs independently, and required assistance from the research therapist prior to TSGT.

Topic areas and themes identified from the daily diaries are summarised in Table 6, and the content analysis in Table 7. Data extracted from daily diaries indicated participant acceptability of interventions.

**Table 6:** Topics and themes identified from the daily diaries.

	**MTS group**	**TI group**
**Topic**	**Themes**	**Themes**
MTS treatment/wearing TIs	Uncomfortable/intense Increased flexibility Better function Benefits	Comfortable Discomfort: - Tightness in shoe - Smooth insole Difficult: Hot weather/swelling Forgot to wear them Awareness of insoles
Changes in feeling in foot/lower limb	Temperature Sensitivity Able to feel foot more Sense of belonging/increased awareness Stability Better movement Foot feels freer	Increased feeling Foot feels ticklish Increased awareness of foot Pain Swelling Temperature
General/comments related to lower limb	Valued treatment Progress Activities of daily living (ADL)	Increased confidence Control Stability
TSGT	Uncomfortable Tiring Challenging Scary Enjoyable Improved balance Improved mobility Ability to walk unaided	Uncomfortable 1st class physio Helpful Enjoyable Balance work/cushion hard Increased confidence Tiring Hard work Challenging Improved movement and balance
Sense of achievement	Increased confidence Increased strength Independence with personal care Ability to walk again Ability to run again Improved balance Returning to normal daily activities: - Car transfers - Kitchen skills - Stairs - Outdoor mobility	Greater independence Better walking Increased movement and flexibility Improved balance Returning to normal daily activities: - Outdoor mobility - Steps - Car transfers
Outcome measures		Tiring

*MTS, Mobilization and tactile stimulation; TI, Textured insoles; TSGT, Task-specific gait training*.

**Table 7:** Content analysis of daily diaries—number of participants for each aspect ticked.

**Aspects ticked in MTS group (*n* = 19)**	**Week 1**	**Week 2**	**Week 3**	**Week 4**	**Week 5**	**Week 6**
Unable to feel as much (in the foot)	1	2	2	1	0	0
Can feel more (in the foot)	15	16	13	11	14	3
MTS was uncomfortable	8 (42.11%)	7 (36.84%)	6 (31.58%)	6 (31.58%)	3 (15.79%)	4 (21.05%)
Discomfort lasted long time	0	0	0	1	1	1
Discomfort did not last long	6	6	6	6	2	4
MTS was not uncomfortable	15 (78.95%)	18 (94.74%)	17 (89.47%)	15 (78.95%)	16 (84.21%)	8 (88.89%)
TSGT was uncomfortable	3 (15.79%)	5 (26.32%)	3 (15.79%)	3 (15.79%)	2 (11.11%)	2 (13.33%)
TSGT was not uncomfortable	18 (94.74%)	17 (89.47%)	18 (94.74%)	19 (100%)	19 (100%)	9 (60%)
Outcome measurements were uncomfortable	0 (0%)	0 (0%)	0 (0%)	0 (0%)	0 (0%)	0 (0%)
Outcome measurements were not uncomfortable	0	6	10	7	3	0
**Aspects ticked in TI group (*****n*** **= 14;** ***n*** **= 13 weeks 2–6)**	**Week 1**	**Week 2**	**Week 3**	**Week 4**	**Week 5**	**Week 6**
Unable to feel as much (in the foot)	1	1	0	0	1	0
Can feel more (in the foot)	7	6	6	6	3	4
Not worn the TIs	1	3	1	2	1	1
Worn TIs <1 h	5	3	2	1	2	1
Worn TIs 2–4 h	3	6	2	3	3	1
Worn TIs more than 5 h	8	11	11	11	12	8
Actual time worn (mean in hours)	6.01	8.05	8.02	7.86	7.72	8.55
TIs uncomfortable	1 (7.14%)	1 (7.69%)	0 (0%)	0 (0%)	0 (0%)	0 (0%)
TIs not uncomfortable	13 (92.86%)	12 (92.31%)	12 (92.31%)	12 (92.31%)	13 (100%)	9 (100%)
TSGT was uncomfortable	0 (0%)	0 (0%)	0 (0%)	1 (7.69%)	1 (7.69%)	1 (11.11%)
TSGT was not uncomfortable	13 (92.86%)	12 (92.31%)	12 (92.31%)	10 (76.92%)	10 (76.92%)	7 (77.78%)
Outcome measurements were uncomfortable	0 (0%)	0 (0%)	0 (0%)	1 (7.69%)	1 (7.69%)	0 (0%)
Outcome measurements were not uncomfortable	3	3	5	2	4	2

*NB: Just the results of the first 6 weeks are presented because this is when the majority of the participants completed the interventions*.

*MTS group: one participant completed the interventions within 4 weeks, nine in 5 weeks, 15 in 6 weeks, 18 in 7 weeks, and one took 9 weeks*.

*TI group: One participant withdrew after 1 week; four participants completed the interventions in 5 weeks, 12 in 6 weeks and all by 7 weeks*.

*MTS, Mobilization and tactile stimulation; TI, Textured insoles; TSGT, Task-specific gait training*.

Four focus groups were held, two for the MTS+TSGT group (focus group 1 *n* = 5; focus group 3 *n* = 8) and two for the TI+TSGT group (focus group 2 *n* = 5; focus group 4 *n* = 2). Of the 32 participants completing the study, 20 (62.50%) attended one of the focus groups: 13 of 19 participants (68.42%) in the MTS+TSGT group, and 7 of 13 participants (53.85%) in the TI+TSGT group.

We specified three main themes *a priori*: (1) acceptability of interventions; (2) acceptability of outcome measures; and (3) overall study experience (see Table 8). We derived sub-themes from the data.

**Table 8:** Final focus group themes summarised, with illustrative quotations.

**Theme**	**Sub-theme**	**Quotations**
Acceptability of the interventions	Comfort/discomfort of somatosensory stimulation interventions	**MTS group:** “*Some parts of it were painful. sticking your thumb in”* (Edward, FG3) “*Painful for the first couple of sessions, after that it was okay”* (Evelyn, FG3) **TI group**: “*I felt surprised actually because when I first saw them I thought “oh dear these are going to be prickly”, and they weren't”* (Olivia, FG 4) “*The textured one on the left foot, when you got used to it, it's more comfortable than the plain one”* (Brian, FG 2) “*Once they was in the shoes they stopped there till I went to bed”* (Isaac, FG 2) “*I transferred it from different shoes that I was wearing, I even put it in my slippers”* (Nadine, FG 2)
	Challenges of TSGT	Tiring: “*I did need to rest afterwards”* (Nancy, FG 3) “*I used to feel tired the next day”* (Phoebe, FG 3)
	Confidence building	**MTS group:** “*My wife isn't the same as you standing there. You do give me confidence.as soon as you dropped off, my confidence dropped”* (Harry, FG 1) “*…gave me the confidence to go out, back out, and to walk with a stick”* (Jackie, FG 3) “*…gave me confidence; you said to me one…one day “you're going to walk now without your stick”…I went from the kitchen into the living room, and I was REALLY surprised at that!”* (Harry, FG 1) “*Well, if it weren't for that treatment I wouldn't be walking now…My wife thinks the same. if it wasn't for you…I wouldn't be walking now, I should be static*” (Harry, FG 1) “*Where I would have gone down…now I find my balance a lot easier.”* (Phoebe, FG 3) **TI group:** “*Having someone who knows what they're talking about, that helps…with confidence*” (Olivia, FG 4) “*It's helped me to achieve my goal and more, um, because I'm now trying things that I wouldn't have tried before.yes, it has absolutely without a doubt. I am even walking, walking on my own now without a walking stick”* (Nadine, FG 2) “*Knowing that I'd got control of my knee meant there was no fear of falling, and I haven't fallen”* (Henry, FG 2) “*Well, life after the trial has been good, because I'm able to get up and move about a bit on my own, transfers…so I'm not a burden on somebody else”* (Henry, FG 2)
Acceptability of the outcome measures	Usefulness of feedback	**MTS group:** “*You showed me the clip of me walking three months ago and I couldn't believe it was me… it had changed dramatically how you're moving; well, one was just moving just through the room and, er it was quite remarkable to see the, the knee and this walking”* (Trevor, FG 1) **TI group:** “*I thought to myself this is measuring the outcome of the progress that I have made and I was quite happy with that”* (Nadine, FG 2) “*You know the pressure sensors that you put in the, er, in the shoes…and then on the computer screen showing equal pressure on both feet I thought that was amazing”* (Henry, FG 2)
	Safety	“*You've got to beware haven't you, of tripping?*” (Edgar, FG 4)
Overall study experience	Intensive therapy at home	**MTS Group:** “*It was ideal. I felt more confident in my own surroundings… it gave you the confidence to do that bit more because you were secure in your surroundings”* (Michelle, FG 3) “*Without you coming to the house, I don't think I would have completed the course because I would think ‘oh, it's too much trouble to go and get ready and go out”’* (Jackie, FG 3) “*I would still have gone* [to the hospital] …*but it's convenient you coming at home”* (Dennis, FG 3) “*I felt it was a bit …stressful, a bit ‘oh no, she's coming again’… for it to be every day it seemed too much”* (Trevor, FG 1) **TI Group:** “*I felt comfortable because it was in my own house and felt as though I'd got more control over it as well”* (Nadine, FG 2) “*Many people are interfering with your life”* (Frank, FG 1)
	Ending the study	**MTS Group:** “*When it all finished I felt like, bloody hell, what am I going to do now?”* (Dennis, FG 3) “*It feels a whole lot better but I'm gutted you've left”* (Evelyn, FG 3) “*I felt sorry it had finished because I felt it was doing me good”* (Jackie, FG 3) **TI Group:** “*After the trial finished…I experienced a sense of loss”* (Nadine, FG 2)

*FG, Focus group; MTS, Mobilization and tactile stimulation; TIs, Textured insoles*.

### Theme 1: Acceptability of Interventions

Three sub-themes were identified: (dis)comfort of the somatosensory stimulation interventions (MTS and TIs); the challenges of TSGT; and confidence building.

#### Sub-theme: Comfort/Discomfort of Somatosensory Stimulation

##### MTS

Content analysis of daily diaries indicated that three participants found aspects of MTS to be uncomfortable or painful, particularly when the massage was deeper, using the thumb, to mobilize tight soft tissues. However, this discomfort was generally described as transient, lasting only a few seconds, and only experienced early in the course of interventions. One participant reported, in the diary, discomfort lasting for a longer period of time, and one other participant noted discomfort that ceased after three or four treatments. Two other participants reported no pain from MTS at all; one participant reported it to be enjoyable.

##### Textured Insoles

Participants found the TIs comfortable to wear, and easy to transfer from shoe to shoe. The textured appearance of the insole caused an initial impression of it being potentially uncomfortable for one participant, although discomfort was not actually experienced when it was worn. Participants generally liked wearing the insoles, and wore them regularly throughout the day, some even wearing them all day. Surprisingly, some participants preferred the sensation of the TI in the contralesional shoe to that of the plain insole in the ipsilesional shoe.

#### Sub-theme: Challenges of Task Specific Gait Training

Whilst TSGT was not reported as being uncomfortable, it was perceived to be challenging, and was described by five participants (from three focus groups) as being hard work. It was also perceived as being tiring, requiring rest or recovery time afterwards, with the fatigue even manifesting itself the following day. TSGT was considered by one participant to be confusing, and two participants described the challenge of TSGT as frightening.

Despite the discomfort and challenges, all three interventions were considered acceptable by participants.

#### Sub-theme: Confidence Building

Interventions in this study were delivered by skilled therapists, and their ability to demonstrate expertise and knowledge was important to participants in terms of building confidence. The presence of an experienced health professional appeared to provide a level of reassurance that waned once the intervention was withdrawn and could not be replicated by the presence of a partner. The experienced therapist provided motivation and instilled a sense of confidence and belief in the ability to walk. This increased confidence and the perceived improvement in mobility was considered by participants to be directly attributable to the intervention, confirming its acceptability. Improvements in balance and lower-limb control were perceived by participants to contribute to this increased confidence and reduced fear of falling and perceived risk of falls.

### Theme 2: Acceptability of Outcome Measures (Objective 4)

Although sometimes challenging, outcome measures were accepted by participants as being an integral component of the study. Overall, the selected outcome measures seemed to have a motivating effect, perhaps making them more acceptable, despite the time required to complete them. Two sub-themes were identified: usefulness of feedback, and safety.

#### Sub-theme: Usefulness of Feedback on Progress

Visual feedback from outcome measures was welcomed by participants, who found it reassuring to see the progress made throughout the study. Video recordings of gait during the modified 5MWT at the start and end of the study were particularly meaningful for one participant to illustrate the quality of his recovering movement and function. On-screen pixelated pressure insole data were of great interest and provided participants with novel insights into weight distribution through each foot in standing.

#### Sub-theme: Safety

The wired Tekscan™ pressure insole set up was a potential safety hazard that had to be managed carefully, particularly during the 5MWT in confined space within the home, to avoid trips over the wires.

### Theme 3: Overall Study Experience (Objectives 3 and 4)

Participants' experiences of the study overall were captured in two sub-themes: intensive therapy at home and ending the study.

#### Sub-theme: Intensive Therapy at Home

Many participants received the interventions in their own home. This was preferable to attending out-patient venues, as they felt more in control and more confident in their own home. It was easier for participants to have the therapist travelling to their house. One participant said she would not have completed the full course of intervention had she been required to travel to receive it, whereas others would have been happy to travel. Regardless, the intensity of mostly daily treatment, often delivered alongside routine NHS therapy, was challenging for some participants.

#### Sub-theme: Ending the Study

By the time the study finished, many participants had developed sufficient movement and ability to undertake functional activities unaided or with their families, and several participants were demonstrating self-management skills. Nevertheless, some participants felt disappointed and let down, when their participation in the study ended, even describing a sense of loss. There was a perception of having made good progress during the study, but a sadness that the intervention had ceased.

### Acceptability and Feasibility of Outcome Measures (Objective 4)

Outcome measures results indicate a decrease in modified 5MWT time from baseline to end of intervention and improvements in the FAC, LEMI, and mRMI scores for both groups, which were maintained at the 1-month follow-up (Table 9).

**Table 9:** Values for each outcome measure at baseline, end of intervention and 1 month follow-up.

**Outcome measure**	**Cost**	**Group**		**Baseline**	**End of intervention**	**1-month follow-up**
Modified 5MWT [seconds]	None	MTS Group		23.80; 26.82 (15.10)^#^	13.43; 16.86 (11.24)	11.41; 16.10 (13.82)
Median; mean (SD)		TI Group		27.65; 35.41 (22.19)^*^	14.51; 21.56 (13.57)^*^	14.79; 21.28 (12.55)^*^
FAC [Scored from 0 to 5]	None	MTS Group		3; 2.37 (1.42)	4; 3.74 (0.87)	4; 4.26 (0.73)
Median; mean (SD)		TI Group		2; 2.00 (1.51)	4; 3.92 (0.86)^*^	4; 4.15 (0.69)^*^
LEMI [Scored from 0 to 100]	None	MTS Group		76; 72.74 (20.99)	86; 83.95 (14.89)	92; 87.26 (16.31)
Median; mean (SD)		TI Group		76; 60.50 (28.79)^#^	84; 75.85 (25.98)^*^	84; 76.01 (23.93)^*^
Ankle ROM [degrees]	Electro-goniometer	MTS group maximum dorsiflexion		9.9; 10.37 (6.01)^*^	9.85; 11.83 (9.02)^#^	8.5; 9.51 (6.27)^#^
Median; mean (SD)	£4,611	TI group maximum dorsiflexion		9.45; 10.05 (5.10)^†^	10.10; 9.67 (3.47)^†^	5.80; 7.57 (3.77)^††^
		MTS group maximum inversion		8.30; 8.48 (3.96)^†^	6.15; 7.78 (4.74)^#^	8.20; 8.26 (4.45)^#^
		TI group maximum inversion		7.0; 7.44 (4.36)^†^	7.70; 9.01 (3.63)^†^	6.10; 7.33 (5.12)^†^
Sensory threshold testing	Semmes	MTS Group	Heel	6; 8.00 (4.14)^#^	10.5; 10.17 (5.22)^#^	10.5; 8.79 (4.45)
(contralesional)	Weinstein		Hallux	10.5; 10.56 (5.06)^#^	10.5; 12.44 (3.99)^#^	11; 10.47 (4.64)
Median; mean (SD)	monofilaments		1st MTP	11; 11.17 (3.55)^#^	12.5; 11.89 (4.75)^#^	11.5; 11.84 (4.31)
[Scored 1–20]	£240		5th MTP	10; 10.32 (4.45)	14.5; 13.39 (4.13)^#^	13; 12.47 (3.95)
		TI Group	Heel	4; 5.23 (3.24)^*^	4; 7.45 (4.63)^††^	4; 6.42 (4.44)^†^
			Hallux	5; 7.25 (4.22)^†^	12; 11.45 (4.39)^††^	9; 9.50 (4.66)^†^
			1st MTP	6; 7.55 (2.94)^††^	10; 10.64 (5.84)^††^	10; 9.50 (6.24)^†^
			5th MTP	7; 7.55 (2.84)^††^	11; 9.42 (5.73)^†^	11; 9.83 (5.17)^†^
Force time integral Median; mean	Pressure insoles £14,000	MTS Group		739.69; 724.43 (317.75)	500.57; 629.07 (280.51)	554.74; 607.34 (284.02)^#^
(SD) [Newtons/sec]		TI Group		635.24; 702.09 (280.84)^#^	636.27; 749.95 (392.18)^*^	707.20; 662.33 (239.32)^*^
Centre of force (AP) velocity		MTS Group		3.60; 4.29 (3.69)	4.70; 5.35 (3.79)	5.60; 5.71 (4.89)^#^
Median; mean (SD) [cm/sec]		TI Group		1.90; 3.30 (3.56)^#^	3.40; 4.05 (3.33)^*^	1.80; 3.84 (3.89)^*^
mRMI [Scored from 0 to 40]		MTS group to ipsilesional side		33; 30.29 (4.83)^*^	34; 33.53 (2.21)^*^	34; 34.22 (0.81)^#^
Median; mean (SD)		TI group to ipsilesional side		23.5; 24.75 (7.62)^†^	34; 32.50 (3.21)^†††^	34; 33.08 (2.15)^†^
		MTS group to contralesional side		33; 29.53 (5.82)^*^	34; 33.47 (3.30)^*^	34; 34.00 (1.11)^#^
		TI group to contralesional side		24.5; 25.25 (7.34)^†^	34; 32.20 (3.58)^†††^	34; 32.75 (2.38)^†^

# *One missing value*,

* *Two missing values*,

† *Three missing values*,

†† *Four missing values*,

††† *Five missing values*.

*AP, Anterior-posterior; FAC, Functional Ambulation Category; LEMI, Lower Extremity Motricity Index; mRMI, modified Rivermead Mobility Index; MTS, Mobilization and tactile stimulation; ROM, Range of movement; SD, Standard deviation; TI, Textured insoles; TSGT, Task-specific gait training; 5MWT, 5 metre walk test*.

A potential ceiling effect was noted for the mRMI results, with 34.21% of the MTS+TSGT group at both end of intervention and 1-month follow-up, and 7.69% of the TI+TSGT group at end of intervention and 26.92% at 1-month follow-up reaching the full 35-point threshold.

During goniometer measurements, which required participants to wear shoes, there was potential for movement of the electrogoniometer within the shoe, affecting reliability of the measurement.

Research assessors reported that measuring touch/pressure sensory thresholds at four sites using the SWMs was very time-consuming. They also reported that setting up, calibrating and recording pressure insole measurements in multiple locations (each participant's home) was both challenging and time-consuming.

In the MTS group no participants stated the outcome measures were uncomfortable, and in the TI+TSGT group only one participant reported that the outcome measures were uncomfortable.

### Success of Blinding

The blinded assessor guessed accurately the group allocation of just three of the 32 participants completing the study: 9.38% (95% CI 3.2%, 24.2%).

### Sample Size Calculation (Objective 5)

The required sample size for a main trial, assuming a minimum clinically important difference of 9.6 m/min on the modified 5MWT (81), 90% power, a two-tailed 5% significance level and equal allocation, would be 51 per group. As small studies may underestimate the standard deviation (82), the observed standard deviation of 12.23 in the current study was inflated by a factor of 1.21 so as to be 90% confident of achieving the nominal power in the main trial (83). Assuming 15% loss to follow up, 60 participants per group would be required.

### Serious Adverse Events, Adverse Events, and Adverse Reactions (Objective 6)

Twenty-seven adverse events were recorded, 14 from the MTS+TSGT group and 13 from the TI+TSGT group. These consisted of: falls (*n* = 16), back pain (*n* = 2), neck pain (*n* = 1), ipsilesional heel pain (*n* = 1), viral infection (*n* = 1), atrial fibrillation (*n* = 1), pressure sore (*n* = 1), scratch on dorsum of foot (*n* = 1), tiredness with swollen painful ankles (*n* = 1), urinary tract infection (*n* = 1), hip and knee pain (*n* = 1). These events were considered by an independent assessor, in accordance with Good Clinical Practice (84) and Standard Operating Procedures for Norwich Clinical Trials Unit and Keele University, and all deemed unrelated to the study. There were no adverse reactions to the interventions or outcome measures. There were three unrelated serious adverse events (one further stroke, and two hospital admissions).

### Pilot Study Aspects to Inform a Future Trial

The traffic light system (78, 79) indicating which aspects of the study should be taken forward is presented in Table 10.

**Table 10:** Planning a future trial: pilot study aspects considered within a traffic light system.

**Traffic light**	**Aspect of pilot study**	**Considerations/changes required**
	Outcome measures	mRMI—Ceiling effect evident and access to stairs issues—not meeting requirements of next trial.
		Ankle range of movement with electrogoniometry—not to be used unless there is further reliability testing. It is inappropriate to measure just the ankle range of movement and not the knee. Use of the tibia to vertical angle (85) may be more clinically useful and could be piloted for further studies.
	Study Management Group	This worked well, but independent steering and data monitoring committees required in future.
	Case report forms	Consider electronic case report forms for future trials.
	Recruitment procedures	Consider involvement of Clinical Research Network to assist in recruitment for future studies.
	Exclusion criteria	Consider whether botulinum toxin should be an exclusion to the trial or not.
	NIHSS	Score four points less for left hemisphere stroke than right; NIHSS useful; staff training is essential.
	Randomization procedures	Consider issue of posterior circulation strokes for randomization in future trial.
	Monitoring length of time wearing TIs	Consider mechanism for recording the length of time TIs are worn if no daily diaries.
	Outcome measures	LEMI—suitable and quick to administer, must specify removal of footwear prior to testing.
		Functional Ambulation Category—Consider access to stairs and inclines for accurate scoring.
		Pressure insoles—Consider purchasing a wireless system to increase the safety of participants and speed of testing. Need to build in time for setting up the equipment and calibrating the insoles.
		Sensory threshold testing with SWMs—Due to the lack of significant results for all but the hallux point in the TI group and time to test, consider applicability for future trials or just test one point.
	Proprioception	Plan to include an outcome measure for assessing proprioception in future trials.
	Patient and public involvement and engagement	The PPIE within MoTaStim-Foot was thorough and beneficial; plan this level of PPIE in future.
	Screening	Step test: This worked well, screening out stroke survivors who functioned at too high a level.
		Ability to follow simple commands screening test: This served its purpose. All participants recruited had capacity to consent and follow therapy instructions.
	Interventions	Thorough training is required for research therapists to ensure adherence all intervention protocols.
	5MWT or 10MWT	Video enabled assessment of assistance to walk/aide. Plan: 1–2 m at start and end of the 5MWT/10MWT.
	Blinded Assessment	This worked well and should be continued for future trials.
	Usual care therapy treatment record	It is important to keep a record of the NHS therapy intervention received.
	Pain/fatigue assessment process and form	This is important to use to monitor pain and fatigue.
	Focus groups exploring participants' perceptions	Hearing participants' opinions is important in a therapy trial because they play an active part in rehabilitation. It also allows for triangulation of methods.
	Focus group schedules	Will need to be adapted as required for a future trial.

*LEMI, Lower Extremity Motricity Index; NIHSS, National Institutes of Health Stroke Scale; mRMI, modified Rivermead Motricity Index; NHS, National Health Service; PPIE, Patient and public involvement and engagement; SWMs, Semmes Weinstein monofilaments; TIs, Textured insoles; 5 or 10MWT, 5 or 10 metre walk test. Red = Should not be retained; Amber = Can be retained with some changes; Green = Can be retained without changes*.

## Discussion

This pilot study estimated the recruitment rate to a subsequent effectiveness trial as 48.57% of stroke survivors screened (objective 1) and the attrition rate as 5.88% (objective 2). The adherence rate to the interventions was 96.88%, ranging between 94.74 and 100%, and the interventions and outcome measures were found to be acceptable to participants (objective 3). The selected outcome measures, which were chosen with all aspects of the ICF in mind, measured sensorimotor impairment and lower-limb function and balance, and were delivered with a successful blinding procedure and were acceptable to all 32 participants who completed the study (objective 4). The estimated variance of outcome measures was used to inform a sample size calculation for a subsequent effectiveness trial (objective 5). No adverse events or reactions that occurred were considered to result from participation in the study (objective 6). Consequently, this pilot study was successful at establishing feasibility of a subsequent pragmatic trial.

The recruitment rate of 48.57% compares well with that of other rehabilitation trials, for example: FAST-INdICATE 5.7% (86); FeSTivaLS 4.6% (87); SWIFT-cast 4.59% (88). However, in our pilot study the number of eligible patients was ascertained from the number referred from the clinical team for screening, and not the total number of stroke patients through the service. In addition, our study included people with posterior circulation as well as anterior circulation stroke. Furthermore, recruitment early post-stroke enabled accrual through hospital services, whereas FeSTivaLS recruited stroke survivors ~2 years after stroke from the community. All of these factors potentially increased the recruitment rate for our pilot study compared with earlier trials.

The attrition rate of 5.88% at both outcome and follow-up also compares favourably with earlier trials; for example, the attrition rate at end of intervention (12.5%) and at follow-up (27.8%) for FAST INdiCATE (86) and end of intervention and follow-up (both 15.5%) in FeSTivaLS (87). As reported, the later recruitment time post-stroke for the FeSTivaLS trial and the longer follow-up time (6 months) in the FAST-INdiCATE trial may have accounted for some of the difference in findings in comparison with our study. The relatively small sample size, and the fact that the study was only undertaken at one site, may also have contributed to this low attrition rate.

An adherence (delivery of interventions to protocol) rate of 96.88% [94.74% (MTS)−100% (TIs)] compares favourably with earlier trials, for example in FeSTivaLS where only 65% of participants completed the full complement of functional strength training for the lower limb. The issues stated above may have contributed to this higher value; however, another key aspect was that in the FeSTivaLS trial each intervention lasted for 1 hour, whereas in MoTaStim-Foot each TSGT session lasted half an hour. Another trial, which also delivered 30 min of task-orientated training to improve lower-limb strength, reported 100% adherence (89).

Acceptability involves many different aspects, including: an ability to understand the intervention; participants' perceived ability to participate (self-efficacy); their perceptions of the intervention and its usefulness; burden, including necessary compromises to enable participation; and congruence of the intervention with the participant's value system (90). Participants in this study confirmed that both the textured and smooth insoles were acceptable and comfortable, and the physical characteristics (density, peak height, and distribution) of the TIs used were acceptable for further study with stroke survivors, with participants able to perceive the TI underfoot and differentiate between a textured and smooth insole. The lack of adverse reactions supports the suitability of using MTS and bespoke TIs as interventions in the future.

Objective 4 included identifying a primary outcome measure for future trials (91). All outcomes were assessed at baseline, end of intervention, and 1-month follow-up, and just the LEMI and SWMs measured more frequently, after 5, 10, and 15 treatments, enabling close monitoring of potential changes to inform intervention dose for a future trial. Selection of the primary and secondary outcome measures for the future trial considered many aspects. This included addressing the different ICF domains, validity, reliability, and responsiveness to change, as well as potential floor or ceiling effects, how feasible it was to undertake the different outcome measures, and their acceptability.

Of the measures used in this trial, we considered the modified 5MWT to be the most appropriate primary outcome measure for a future trial: it is reliable (92), responsive to change in walking ability after stroke (58), clinically relevant, quick and easy to set up, and demonstrated potential for change. It provides ratio-level data, has no ceiling or floor effect limitations, and incurs negligible costs. However, to enable improved standardisation of procedure for future trials, a larger space in an appropriate location, such as a university laboratory or hospital setting, should be used rather than the limited space in participants' homes, which does not allow for the recommended 2–3 m (55, 92) for acceleration and deceleration. Further consideration will also be given to the 10MWT or 6-min walk, as advocated by the Stroke Recovery and Rehabilitation Roundtable guidelines for stroke research (66).

For secondary outcome measures to take forward, we selected the LEMI, which is a valid and reliable measure of motor impairment (56, 57) and responsive to change (Table 9), and the FAC, also valid and reliable for classifying functional ability for a stroke population (52), quick to undertake, and responsive to change (Table 9).

A ceiling effect was evident from the mRMI data (93), with median scores of 34 (out of a maximum of 35) in each of the categories at end of intervention and follow-up for both groups. Restricted access to or absence of stairs in participants' homes was an additional limitation of the mRMI. Consequently, we did not consider the mRMI to be a suitable outcome measure for a larger trial.

We found that potential movement of the electrogoniometer within the shoe may have affected reliability and validity of the goniometry data. Whilst this has not been identified as a potential issue by the manufacturer, further investigation of reliability needs to be done before including this in a larger trial.

Touch/pressure sensory threshold testing at four different sites on the plantar surface of the foot was time-consuming. Repeated testing has potential either to increase testing fatigue and reduce attention to the task and, therefore, reliability, or to enhance sensibility of subsequent testing sites by raising awareness of the foot as a whole. Despite using a bespoke algorithm for SWM testing in this study (Supplementary Material), which decreased the burden of testing for participants by reducing the number of filaments used at each location, we need to consider further the choice and number of testing sites. Testing just one site may be sufficient, particularly if that one site indicates either fully intact or absent sensation (94); if sensation at one site is affected, it is likely that all sites will be affected, due to stroke being an upper motor neurone lesion.

Furthermore, assessment of cutaneous touch/pressure thresholds from the plantar surface of the foot in a non-weight-bearing position may not appropriately reflect afferent information received in an upright standing posture. Afferent information from ankle proprioceptors may decrease the importance of cutaneous afferent information from the plantar surface of the foot during balance (95). Impaired proprioception post-stroke is more prevalent than tactile sensory impairment (96) and alignment of the foot and ankle is important to enable normal balance and gait. Although MTS provides tactile information through the cutaneous afferents, an important aim is to provide intensive proprioceptive stimulation (12). Consequently, measurement of proprioception, using the Erasmus version of the Nottingham Sensory Assessment or the sensory section of the Fugl–Meyer Assessment, advocated with careful standardised testing procedures (68), will be considered prior to the next trial.

The challenges relating to setting up and calibrating pressure insoles should be factored into planning the burden of assessment if these insoles are to be included in future studies. Extreme care was necessary to ensure participants' safety, due to the long wires; a wireless system is advised. The additional cost implications need to be considered.

The perceived sense of loss reported in relation to termination of the study needs to be borne in mind for subsequent rehabilitation trials; participants should be adequately supported.

### Limitations of This Pilot Study

Undertaking this study at one site, which had been involved in other trials involving MTS and other therapy interventions, may have introduced bias through prior familiarity with the intervention and the research team.

The sample size for our study was the largest achievable within available resources, and whilst smaller than that recommended for estimates of binary outcomes (97), it was broadly in line with recommendations for continuous outcomes (40, 41).

### Strengths of This Pilot Study

The sample was representative of the stroke population for age, gender, and type of stroke when comparing with other rehabilitation trials (86–88); the small sample size would account for slight discrepancies.

The study followed the guidance in the CONSORT 2010 statement extension to randomized pilot and feasibility trials (75, 98) and the psychometric properties of the outcome measures informed their selection. Research therapists and assessors were appropriately trained, and standardised procedures and protocols were followed for delivering interventions and undertaking outcome measures. Data collection triangulation, by including both daily diaries and focus groups, increased credibility within the study (99).

The value of PPIE in health research has been increasingly recognised, with the NIHR and stroke research networks embracing strategies to increase PPIE input (100). Involvement of PPIE volunteers at all stages of the MoTaSTim-Foot pilot study from initial stages through to dissemination at the UK Stroke Forum, with involvement in almost every aspect of the study, was an important strength. The inclusion of an additional member of the research team (a stroke survivor, a PPIE volunteer) as observer and field-note taker during the focus groups enabled additional insights and an appreciation of the context behind the participants' dialogue, reducing the potential bias associated with a single investigator (101).

## Conclusion

This study has successfully provided feasibility information in preparation for a subsequent RCT, suggesting that the subsequent trial will have a high recruitment and low attrition rate, excellent adherence to the experimental and control interventions, and that these interventions and outcome measures will be acceptable to stroke survivors with no expected adverse reactions. We can conclude that a larger RCT is feasible and should have a nominal sample size of 60 participants per group based on the modified 5MWT as the primary outcome measure.

However, prior to undertaking a definitive clinical trial, further consideration will be given to whether the trial can be delivered successfully in other regions, the optimal dose of intervention and most appropriate target population and time post stroke, in accordance with the second Stroke Recovery and Rehabilitation Roundtable recommendations (53).

## Data Availability Statement

The raw data supporting the conclusions of this article will be made available by the authors, without undue reservation.

## Ethics Statement

The studies involving human participants were reviewed and approved by UK National Research Ethics Service (4/3/16), IRAS No: 171968/REC Ref 16/WM/0080. The patients/participants provided their written informed consent to participate in this study.

## Author Contributions

AMA, JS, SMH, and VMP developed the statistical analysis plan. AMA and SMH checked the quantitative database for accuracy, exported the data for statistical analysis, analyzed the daily diaries, and drafted the manuscript. AMA, SMH, and JS performed the statistical analyses. AMA, SMH, and SR undertook the thematic analysis of the focus groups. AMA was Chief Investigator for the MoTaStim-Foot pilot study, with supervision from SMH, VMP, JS, and SR. All authors revised the manuscript for important intellectual content, read and approved the final manuscript, and participated in the design of the study.

## Conflict of Interest

The authors declare that the research was conducted in the absence of any commercial or financial relationships that could be construed as a potential conflict of interest.

## Footnotes

^1^Algeos UK Ltd (http://www.algeos.com/).

^2^http://www.nihstrokescale.org/

^3^http://www.biometricsltd.com/gonio.htm

## Abbreviations

5MWT: Five metre walk test
LEMI: Lower Extremity Motricity Index
MoTaStim-Foot: Mobilization and Tactile Stimulation of the Foot
mRMI: Modified Rivermead Mobility Index
MTS: Mobilization and tactile stimulation
NIHSS: National Institutes of Health Stroke Scale
PPIE: Patient and public involvement and engagement
SWMs: Semmes Weinstein monofilaments
TI: Textured insole
TSGT: Task-specific gait training.
CI: Confidence interval
IQR: Interquartile range
NHS: National Health Service
SD: Standard deviation
UK: United Kingdom.

## References

[1] Stroke-Association. State of the Nation - Stroke Statistics. Stroke Association (2017). Available online at: https://www.stroke.org.uk/sites/default/files/state_of_the_nation_2017_final_1.pdf; https://www.stroke.org.uk/resources/state-nation-stroke-statistics?gclid=EAIaIQobChMIkIiowMC94gIVrLftCh1sAwEnEAAYASAAEgJUHvD_BwE

[2] Feigenson JS McCarthy ML Meese PD Feigenson WD Greenberg SD Rubin E. Stroke rehabilitation I. Factors predicting outcome and length of stay-an overview. N Y State J Med. (1977) 77:1426–30. 267835

[3] Kim JS Choi-Kwon S. Discriminative sensory dysfunction after unilateral stroke. Stroke. (1996) 27:677–82. 10.1161/01.STR.27.4.677 8614929

[4] Carey LM. Stroke Rehabilitation Insights From Neuroscience and Imaging. Oxford: Oxford University Press (2012).

[5] Tyson SF Crow JL Connell L Winward C Hillier S. Sensory impairments of the lower limb after stroke: a pooled analysis of individual patient data. Topics Stroke Rehabil. (2013) 20:441–9. 10.1310/tsr2005-441 24091286

[6] Gorst T Rogers A Morrison SC Cramp M Paton J Freeman J. The prevalence, distribution, and functional importance of lower limb somatosensory impairments in chronic stroke survivors: a cross sectional observational study. Disabil Rehabil. (2018) 41:2433–50. 10.1080/09638288.2018.1468932 29726732

[7] Patel AT Duncan PW Lai S Studenski S. The relation between impairments and functional outcomes poststroke. Arch Phys Med Rehabil. (2000) 81:1357–63. 10.1053/apmr.2000.9397 11030501

[8] Sánchez-Blanco I Ochoa-Sangrador C López-Munain L Izquierdo-Sánchez M Fermoso-Garcia J. Predictive model of functional independence in stroke patients admitted to a rehabilitation programme. Clin Rehabil. (1999) 13:464–75. 10.1191/026921599672994947 10588532

[9] Pollock A St George B Fenton M Firkins L. Top 10 research priorities relating to life after stroke–consensus from stroke survivors, caregivers, and health professionals. Int J Stroke. (2014) 9:313–20. 10.1111/j.1747-4949.2012.00942.x 23227818

[10] Bernhardt J Hayward KS Kwakkel G Ward NS Wolf SL Borschmann K. Agreed definitions and a shared vision for new standards in stroke recovery research: the stroke recovery and rehabilitation roundtable taskforce. Int J Stroke. (2017) 12:444–50. 10.1177/1747493017711816 28697708

[11] Pomeroy V Aglioti SM Mark VW McFarland D Stinear C Wolf SL. Neurological principles and rehabilitation of action disorders: rehabilitation interventions. Neurorehabil Neural Repair. (2011) 25:33S−43S. 10.1177/1545968311410942 21613536 PMC4139494

[12] Hunter SM Crome P Sim J Donaldson C Pomeroy VM. Development of treatment schedules for research: a structured review to identify methodologies used and a worked example of ‘mobilisation and tactile stimulation’ for stroke patients. Physiotherapy. (2006) 92:195–207. 10.1016/j.physio.2006.01.001

[13] Hunter SM Crome P Sim J Pomeroy VM. Effects of mobilization and tactile stimulation on recovery of the hemiplegic upper limb: a series of replicated single-system studies. Arch Phys Med Rehabil. (2008) 89:2003–10. 10.1016/j.apmr.2008.03.016 18929030

[14] Winter JM Crome P Sim J Hunter SM. Effects of mobilization and tactile stimulation on chronic upper-limb sensorimotor dysfunction after stroke. Arch Phys Med Rehabil. (2013) 94:693–702. 10.1016/j.apmr.2012.11.028 23201425

[15] Hillier S Dunsford A. A pilot study of sensory retraining for the hemiparetic foot post-stroke. Int J Rehabil Res. (2006) 29:237–42. 10.1097/01.mrr.0000210052.32539.22 16900045

[16] Aries AM Cooke L Hunter S. Mobilization and tactile (sensory) stimulation (mts) for the foot post stroke: opinions and perceptions of expert clinicians. Int J Ther Rehabil. (2019) 26:15. 10.12968/ijtr.2019.26.6.15

[17] Orth D Davids K Wheat J Seifert L Liukkonen J Jaakkola T. The role of textured material in supporting perceptual-motor functions. PLoS ONE. (2013) 8:e60349. 10.1371/journal.pone.0060349 23565232 PMC3615024

[18] Christovão T Neto H Grecco L Ferreira L de Moura R de Souza M. Effect of different insoles on postural balance: a systematic review. J Phys Ther Sci. (2013) 25:1353–6. 10.1589/jpts.25.1353 24259792 PMC3820199

[19] Cruz-Almeida Y Black ML Christou EA Clark DJ. Site-specific differences in the association between plantar tactile perception and mobility function in older adults. Front Aging Neurosci. (2014) 6:68. 10.3389/fnagi.2014.00068 24782765 PMC3990110

[20] Kavounoudias A Roll R Roll J-P. The plantar sole is a 'dynamometric map' for human balance control. Neuroreport. (1998) 9:3247–52. 10.1097/00001756-199810050-00021 9831459

[21] Ribot-Ciscar E Vedel JP Roll JP. Vibration sensitivity of slowly and rapidly adapting cutaneous mechanoreceptors in the human foot and leg. Neurosci Lett. (1989) 104:130–5. 10.1016/0304-3940(89)90342-X 2812525

[22] Smania N Montagnana B Faccioli S Fiaschi A Aglioti SM. Rehabilitation of somatic sensation and related deficit of motor control in patients with pure sensory stroke. Arch Phys Med Rehabil. (2003) 84:1692–702. 10.1053/S0003-9993(03)00277-6 14639572

[23] Raine S Meadows L Lynch-Ellerington M. Bobath Concept: Theory and Clinical Practice in Neurological Rehabilitation. Chichester: Blackwell Publishing (2009).

[24] Hatton AL Dixon J Rome K Martin D. Standing on textured surfaces: effects on standing balance in healthy older adults. Age Ageing. (2011) 40:363–8. 10.1093/ageing/afr026 21450692

[25] Dixon J Hatton AL Robinson J Gamesby-Iyayi H Hodgson D Rome K. Effect of textured insoles on balance and gait in people with multiple sclerosis: an exploratory trial. Physiotherapy. (2014) 100:142–9. 10.1016/j.physio.2013.06.003 24070573

[26] Veerbeek JM van Wegen E van Peppen R van der Wees PJ Hendriks E Rietberg M. What is the evidence for physical therapy poststroke? A systematic review and meta-analysis. PLoS ONE. (2014) 9:e87987. 10.1371/journal.pone.0087987 24505342 PMC3913786

[27] French B Thomas LH Coupe J McMahon NE Connell L Harrison J. Repetitive task training for improving functional ability after stroke. Cochrane Database Syst Rev. (2016) 11:CD006073. 10.1002/14651858.CD006073.pub3 27841442 PMC6464929

[28] Rudd A Bowen A Young G James M. The latest national clinical guideline for stroke: 5th edition. Clin Med. (2017) 17:154–5. 10.7861/clinmedicine.17-2-154 PMC6297617 28365628

[29] Salbach NM Mayo NE Wood-Dauphinee S Hanley JA Richards CL Côté R. A task-orientated intervention enhances walking distance and speed in the first year post stroke: a randomized controlled trial. Clin Rehabil. (2004) 18:509–19. 10.1191/0269215504cr763oa 15293485

[30] Dean CM Richards CL Malouin F. Task-related circuit training improves performance of locomotor tasks in chronic stroke: a randomized, controlled pilot trial. Arch Phys Med Rehabil. (2000) 81:409–17. 10.1053/mr.2000.3839 10768528

[31] French B Leathley M Sutton C McAdam J Thomas L Forster A. A systematic review of repetitive functional task practice with modelling of resource use, costs and effectiveness. Health Technol Assess. (2008) 12:iii. 10.3310/hta12300 18547501

[32] Rossignol S Dubuc R Gossard J-P. Dynamic sensorimotor interactions in locomotion. Physiol Rev. (2006) 86:89–154. 10.1152/physrev.00028.2005 16371596

[33] Laaksonen K Kirveskari E Mäkelä JP Kaste M Mustanoja S Nummenmaa L. Effect of afferent input on motor cortex excitability during stroke recovery. Clin Neurophysiol. (2012) 123:2429–36. 10.1016/j.clinph.2012.05.017 22721651

[34] Craig P Dieppe P Macintyre S Michie S Nazareth I Petticrew M. Developing and Evaluating Complex Interventions: New Guidance. (2006). Available online at: https://mrc.Ukri.Org/documents/pdf/complex-interventions-guidance/ (accessed February 2, 2021).

[35] Langhorne P Bernhardt J Kwakkel G. Stroke rehabilitation. Lancet. (2011) 377:1693–702. 10.1016/S0140-6736(11)60325-5 21571152

[36] Alexander MP. Stroke rehabilitation outcome. A potential use of predictive variables to establish levels of care. Stroke. (1994) 25:128–34. 10.1161/01.STR.25.1.128 8266360

[37] Clark L Fairhurst C Torgerson DJ. Allocation concealment in randomised controlled trials: are we getting better? BMJ. (2016) 355:i5663. 10.1136/bmj.i5663 27856428

[38] Hill KD Bernhardt J McGann AM Maltese D Berkovits D. A new test of dynamic standing balance for stroke patients: reliability, validity and comparison with healthy elderly. Physiother Can. (1996) 48:257–62. 10.3138/ptc.48.4.257

[39] Thabane L Ma J Chu R Cheng J Ismaila A Rios LP. A tutorial on pilot studies: the what, why and how. BMC Med Res Methodol. (2010) 10:1. 10.1186/1471-2288-10-1 20053272 PMC2824145

[40] Browne RH. On the use of a pilot sample for sample size determination. Stat Med. (1995) 14:1933–40. 10.1002/sim.4780141709 8532986

[41] Pomeroy VM Cooke E Hamilton S Whittet A Tallis RC. Development of a schedule of current physiotherapy treatment used to improve movement control and functional use of the lower limb after stroke: a precursor to a clinical trial. Neurorehabil Neural Repair. (2005) 19:350–9. 10.1177/1545968305280581 16263967

[42] Aries AM. Somatosensory stimulation to improve lower-limb recovery after stroke (PhD Thesis). Keele University, Newcastle-under-Lyme, Staffordshire (2020).

[43] Hoffmann TC Glasziou PP Boutron I Milne R Perera R Moher D. Better reporting of interventions: template for Intervention Description and Replication (TIDieR) checklist and guide. Gesundheitswesen. (2016) 78:175–88. 10.1136/bmj.g1687 26824401

[44] O'Cathain A Croot L Duncan E Rousseau N Sworn K Turner KM. Guidance on how to develop complex interventions to improve health and healthcare. BMJ Open. (2019) 9:e029954. 10.1136/bmjopen-2019-029954 31420394 PMC6701588

[45] Hunter SM Hammett L Ball S Smith N Anderson C Clark A. Dose-response study of mobilisation and tactile stimulation therapy for the upper extremity early after stroke: a phase 1 trial. Neurorehabil Neural Repair. (2011) 25:314–22. 10.1177/1545968310390223 21282528

[46] Ballinger C Ashburn A Low J Roderick P. Unpacking the black box of therapy—a pilot study to describe occupational therapy and physiotherapy interventions for people with stroke. Clin Rehabil. (1999) 13:301–9. 10.1191/026921599673198490 10460118

[47] Watanabe I Okubo J. The role of the plantar mechanoreceptor in equilibrium control. Ann N Y Acad Sci. (1981) 374:855–64. 10.1111/j.1749-6632.1981.tb30926.x 6951463

[48] Kennedy PM Inglis JT. Distribution and behaviour of glabrous cutaneous receptors in the human foot sole. J Physiol. (2002) 538:995–1002. 10.1113/jphysiol.2001.013087 11826182 PMC2290100

[49] Corbin DM Hart JM Palmieri-Smith R Ingersoll CD Hertel J. The effect of textured insoles on postural control in double and single limb stance. J Sport Rehabil. (2007) 16:363–72. 10.1123/jsr.16.4.363 18246902

[50] Wiener J Foley N Peireira S Cotoi A Chow J Janssen S. Mobility and the Lower Extremity. Ebrsr: Evidence-Based Review of stroke Rehabilitation, Review of Stroke Rehabilitation. (2018). p. 1–191. Available online at: http://www.Ebrsr.Com/evidence-review/9-mobility-and-lower-extremity (accessed April 30, 2021).

[51] Meyer BC Lyden PD. The modified national institutes of health stroke scale: its time has come. Int J Stroke. (2009) 4:267–73. 10.1111/j.1747-4949.2009.00294.x 19689755 PMC2729912

[52] Mehrholz J Wagner K Rutte K Meissner D Pohl M. Predictive validity and responsiveness of the functional ambulation category in hemiparetic patients after stroke. Arch Phys Med Rehabil. (2007) 88:1314–9. 10.1016/j.apmr.2007.06.764 17908575

[53] Bernhardt J Hayward KS Dancause N Lannin NA Ward NS Nudo RJ. A stroke recovery trial development framework: consensus-based core recommendations from the second stroke recovery and rehabilitation roundtable. Neurorehabil Neural Repair. (2019) 33:959–69. 10.1177/1545968319888642 31674274

[54] Bronner S Agraharasamakulam S Ojofeitimi S. Reliability and validity of a new ankle electrogoniometer. J Med Eng Technol. (2010) 35:350–5. 10.3109/03091902.2010.493968 20586555

[55] Tracey EH Greene AJ Doty RL. Optimizing reliability and sensitivity of semmes–weinstein monofilaments for establishing point tactile thresholds. Physiol Behav. (2012) 105:982–6. 10.1016/j.physbeh.2011.11.002 22100625

[56] Cameron D Bohannon RW. Criterion validity of lower extremity motricity index scores. Clin Rehabil. (2000) 14:208–11. 10.1191/026921500675786655 10763800

[57] Fayazi M Dehkordi SN Dadgoo M Salehi M. Test-retest reliability of motricity index strength assessments for lower extremity in post stroke hemiparesis. Med J Islamic Republic Iran. (2012) 26:27–30. 23483112 PMC3587895

[58] Salbach NM Mayo NE Higgins J Ahmed S Finch LE Richards CL. Responsiveness and predictability of gait speed and other disability measures in acute stroke. Arch Phys Med Rehabil. (2001) 82:1204–12. 10.1053/apmr.2001.24907 11552192

[59] Low DC Dixon SJ. Footscan pressure insoles: accuracy and reliability of force and pressure measurements in running. Gait Posture. (2010) 32:664–6. 10.1016/j.gaitpost.2010.08.002 20813530

[60] Chesnin KJ Selby-Silverstein L Besser MP. Comparison of an in-shoe pressure measurement device to a force plate: concurrent validity of center of pressure measurements. Gait Posture. (2000) 12:128–33. 10.1016/S0966-6362(00)00071-0 10998609

[61] Lennon S Johnson L. The modified rivermead mobility index: validity and reliability. Disabil Rehabil. (2000) 22:833–9. 10.1080/09638280050207884 11197520

[62] Krueger RA Casey MA. Focus Groups: A Practical Guide for Applied Research. Thousand Oaks, CA: Sage Publications, Inc. (2000).

[63] Braun V Clarke V. Using thematic analysis in psychology. Qual Res Psychol. (2006) 3:77–101. 10.1191/1478088706qp063oa

[64] World Health Organization (WHO). How to Use the ICF: A Practical Manual for Using the International Classification of Functioning, Disability and Health (ICF). Exposure draft for comment. Geneva: WHO (2013). Available online at: https://cdn.who.int/media/docs/default-source/classification/icf/drafticfpracticalmanual2.pdf?sfvrsn=8a214b01_4 (accessed April 24, 2021).

[65] Sim J Wright C. Research in Health Care: Concepts, Designs and Methods. Cheltenham: Stanley Thornes (2000).

[66] Kwakkel G Lannin NA Borschmann K English C Ali M Churilov L. Standardized measurement of sensorimotor recovery in stroke trials: consensus-based core recommendations from the stroke recovery and rehabilitation roundtable. Neurorehabil Neural Repair. (2017) 31:784–92. 10.1177/1545968317732662 28934918

[67] Moriguchi CS Sato TO Coury HJCG. Ankle movements during normal gait evaluated by flexible electrogoniometer. / Movimentos do tornozelo durante a marcha normal avaliados por eletrogoniometria flexível. Brazil J Phys Ther. (2007) 11:205–11. 10.1590/S1413-35552007000300006

[68] Connell LA Tyson SF. Measures of sensation in neurological conditions: a systematic review. Clin Rehabil. (2012) 26:68–80. 10.1177/0269215511412982 21971756

[69] Suzuki K Nakamura R Yamada Y Handa T. Determinants of maximum walking speed in hemiparetic stroke patients. Tohoku J Exp Med. (1990) 162:337–44. 10.1620/tjem.162.337 2102565

[70] Jacelon CS Imperio K. Participant diaries as a source of data in research with older adults. Qual Health Res. (2005) 15:991–7. 10.1177/1049732305278603 16093375

[71] McMahon SA Winch PJ. Systematic debriefing after qualitative encounters: an essential analysis step in applied qualitative research. BMJ Global Health. (2018) 3:e000837. 10.1136/bmjgh-2018-000837 30233833 PMC6135453

[72] Fischer CT. Bracketing in qualitative research: conceptual and practical matters. Psychother Res. (2009) 19:583–90. 10.1080/10503300902798375 20183407

[73] Copeland D Liska H. Implementation of a post-code pause: extending post-event debriefing to include silence. J Trauma Nurs. (2016) 23:58–64. 10.1097/JTN.0000000000000187 26953532

[74] Sim J. Should treatment effects be estimated in pilot and feasibility studies? Pilot Feasibility Stud. (2019) 5:107. 10.1186/s40814-019-0493-7 31485336 PMC6712606

[75] Eldridge SM Chan CL Campbell MJ Bond CM Hopewell S Thabane L. Consort 2010 statement: extension to randomised pilot and feasibility trials. BMJ. (2016) 355:1–29. 10.1186/s40814-016-0105-8 27777223 PMC5076380

[76] Krippendorff K. Content Analysis: An Introduction to Its Methodology, 3rd ed. Thousand Oaks, CA: Sage Publications (2013).

[77] Ziebland S McPherson A. Making sense of qualitative data analysis: an introduction with illustrations from dipex (personal experiences of health and illness). Med Educ. (2006) 40:405–14. 10.1111/j.1365-2929.2006.02467.x 16635119

[78] ICO. Guide to the General Data Protection Regulation (GDPR). Information Commissioner's Office (2020). Available online at: https://ico.org.uk/for-organisations/guide-to-data-protection/guide-to-the-general-data-protection-regulation-gdpr/ (accessed February 2, 2021).

[79] Tilson JK Sullivan KJ Cen SY Rose DK Koradia CH Azen SP. Meaningful gait speed improvement during the first 60 days poststroke: minimal clinically important difference. Phys Ther. (2010) 90:196–208. 10.2522/ptj.20090079 20022995 PMC2816032

[80] Vickers AJ. Underpowering in randomized trials reporting a sample size calculation. J Clin Epidemiol. (2003) 56:717–20. 10.1016/S0895-4356(03)00141-0 12954462

[81] Sim J Lewis M. The size of a pilot study for a clinical trial should be calculated in relation to considerations of precision and efficiency. J Clin Epidemiol. (2012) 65:301–8. 10.1016/j.jclinepi.2011.07.011 22169081

[82] Bhatt A. International council for harmonisation e6(r2) addendum: challenges of implementation. Perspect Clin Res. (2017) 8:162–6. 10.4103/picr.PICR_124_17 29109932 PMC5654214

[83] Sheron N Moore M Ansett S Parsons C Bateman A. Developing a 'traffic light' test with potential for rational early diagnosis of liver fibrosis and cirrhosis in the community. Br J General Prac. (2012) 62:616–24. 10.3399/bjgp12X654588 22947582 PMC3426600

[84] Meijel B Hamersveld S Gool R der Bijl J Harten P. Effects and feasibility of the 'traffic light method for somatic screening and lifestyle' in patients with severe mental illness: a pilot study. Perspect Psychiatric Care. (2015) 51:106–13. 10.1111/ppc.12071 24735008

[85] Kerr A Rowe P Clarke A Chandler E Smith J Ugbolue C. Biomechanical correlates for recovering walking speed early after stroke. Is the tibia to vertical angle a distinctive therapy target? Gait Posture. (2019) 73:277–8. 10.1016/j.gaitpost.2019.07.150 31862664

[86] Hunter SM Johansen-Berg H Ward N Kennedy NC Chandler E Weir CJ. Functional strength training and movement performance therapy for upper limb recovery early poststroke-efficacy, neural correlates, predictive markers, and cost-effectiveness: fast-indicate trial. Front Neurol. (2018) 8:733. 10.3389/fneur.2017.00733 29472884 PMC5810279

[87] Mares K Cross J Clark A Vaughan S Barton GR Poland F. Feasibility of a randomized controlled trial of functional strength training for people between six months and five years after stroke: festivals trial. Trials. (2014) 15:1–11. 10.1186/1745-6215-15-322 25118156 PMC4138387

[88] Pomeroy VM Rowe P Clark A Walker A Kerr A Chandler E. A randomized controlled evaluation of the efficacy of an ankle-foot cast on walking recovery early after stroke: swift cast trial. Neurorehabil Neural Repair. (2016) 30:40–8. 10.1177/1545968315583724 25931239 PMC4704299

[89] Yang YR Wang RY Lin KH Chu MY Chan RC. Task-oriented progressive resistance strength training improves muscle strength and functional performance in individuals with stroke. Clin Rehabil. (2006) 20:860–70. 10.1177/0269215506070701 17008338

[90] Sekhon M Cartwright M Francis JJ. Acceptability of healthcare interventions: an overview of reviews and development of a theoretical framework. BMC Health Serv Res. (2017) 17:88. 10.1186/s12913-017-2031-8 28126032 PMC5267473

[91] Lancaster GA Dodd S Williamson PR. Design and analysis of pilot studies: recommendations for good practice. J Eval Clin Prac. (2004) 10:307–12. 10.1111/j.0.2002.384.doc.x 15189396

[92] Collen FM Wade DT Bradshaw CM. Mobility after stroke: reliability of measures of impairment and disability. Int Disabil Stud. (1990) 12:6–9. 10.3109/03790799009166594 2211468

[93] Johnson L Selfe J. Measurement of mobility following stroke: a comparison of the modified rivermead mobility index and the motor assessment scale. Physiotherapy. (2004) 90:132–8. 10.1016/j.physio.2004.01.004

[94] Busse M Tyson SF. How many body locations need to be tested when assessing sensation after stroke? An investigation of redundancy in the rivermead assessment of somatosensory performance. Clin Rehabil. (2009) 23:91–5. 10.1177/0269215508097296 19114441

[95] Marigold DS Eng JJ Tokuno CD Donnelly CA. Contribution of muscle strength and integration of afferent input to postural instability in persons with stroke. Neurorehabil Neural Repair. (2004) 18:222–39. 10.1177/1545968304271171 15537993 PMC3226790

[96] Connell LA Lincoln NB Radford KA. Somatosensory impairment after stroke: frequency of different deficits and their recovery. Clin Rehabil. (2008) 22:758–67. 10.1177/0269215508090674 18678576

[97] Teare MD Dimairo M Shephard N Hayman A Whitehead A Walters SJ. Sample size requirements to estimate key design parameters from external pilot randomised controlled trials: a simulation study. Trials. (2014) 15:264. 10.1186/1745-6215-15-264 24993581 PMC4227298

[98] Moher D Hopewell S Schulz KF Montori V Gøtzsche PC Devereaux PJ. Consort 2010 explanation and elaboration: updated guidelines for reporting parallel group randomised trials. Int J Surg. (2012) 10:28–55. 10.1016/j.ijsu.2011.10.001 22036893

[99] Nowell LS Norris JM White DE Moules NJ. Thematic analysis: striving to meet the trustworthiness criteria. Int J Qualitative Methods. (2017) 16:1. 10.1177/1609406917733847

[100] Ardron D Kendall M. Patient and public involvement in health research: what is it, and why is it so important? Int J Palliative Nurs. (2010) 16:160–2. 10.12968/ijpn.2010.16.4.47778 20559177

[101] Archibald MM. Investigator triangulation: a collaborative strategy with potential for mixed methods research. J Mixed Methods Res. (2016) 10:228–50. 10.1177/1558689815570092

