# Supplementary material for: Sensory Stimulation of the Foot and Ankle Early Post-stroke: A Pilot and Feasibility Study
Source: Front Neurol. 2021 Jul 5;12:675106. doi: 10.3389/fneur.2021.675106 (PMC8287025; doi:10.3389/fneur.2021.675106)
Supplement: Supplementary file 1 [file Data_Sheet_1.pdf]

Sensory stimulation of the foot and ankle early post-stroke: a pilot and feasibility study

Supplementary information 1. Textured insole (TI) group daily diary

Supplementary information 2. Mobilization and tactile stimulation (MTS) daily diary

Supplementary information 3. Record of task-specific gait training (TSGT) delivered

Supplementary information 4. Bespoke algorithm for Semmes Weinstein monofilament (SWM) sensory threshold testing

Supplementary information 5a. Focus group schedule for the mobilization and tactile stimulation (MTS) groups

Supplementary information 5b. Focus group schedule for the textured insole (TI) groups

frontiers

Supplementary information 1: Textured insole (TI) group daily diary

Week Commencing: _ _ _ _ . _ _ _ _ (DD-MM-YYYY)
Screening Number: ______________

Site Code: _______
Participant number: _________

Staffordshire and NHS
Stoke on Trent Partnership
NHS Trust

DAILY DIARY

Study Title: Sensory stimulation of the foot and ankle early post stroke: a feasibility study (MoTaStim –Foot)

Name of researcher: ______________________________ Name of participant: ______________________________

WEEK BEGINNING:	Sun	Mon	Tue	Wed	Thur	Fri	Sat
**1. The Feeling within your foot:** (Please tick any that apply to you)							
1a)Today my foot feels cold							
1b)Today my foot feels warm							
1c) Today my foot felt sensitive							
1d) Today my foot did not feel sensitive							
1e) There is no change in my foot at all →							
1f) Today I am unable to feel as much in my foot ↓							
1g) Today I can feel my foot more ↑							
**2. Wearing the textured insoles** (TIs)							
2a) Today I have not worn my TIs							
2a) Today I have worn my TIs for less than one hour							
2b) Today I have worn my TIs for between 2-4 hours							
2c) Today I have worn my TIs for more than 5 hours							

Week Commencing: _ _ _ _ . _ _ _ _ (DD-MM-YYYY)
Screening Number: ______________

Site Code: _______
Participant number: _________

**3. About your treatments:** (Please tick any that apply to you)	Sun	Mon	Tue	Wed	Thur	Fri	Sat
3a) Today wearing my TIs was uncomfortable							
3b) Today wearing my TIs was **NOT** uncomfortable							
3c) Today the task specific walking training was uncomfortable							
3d) Today the task specific walking training was **NOT** uncomfortable							
3e) Today the outcome measurements were uncomfortable							
3f) Today the outcome measurements were **NOT** uncomfortable							
Additional comments:							

Supplementary information 2: Mobilization and tactile stimulation (MTS) group daily diary

Week commencing: _ _-_ _-_ _ _ _ (DD-MM-YYYY)
Screening Number: _______________

Site Code: _______
Participant Number: ______

Staffordshire and Stoke on Trent Partnership NHS Trust

DAILY DIARY

Study Title: Mobilization and sensory stimulation of the foot and ankle post stroke: a feasibility study

Name of researcher: ______________________ Name of participant: ______________________

WEEK BEGINNING: (Please tick any that apply to you)	Sun	Mon	Tue	Wed	Thur	Fri	Sat
1. **The Feeling within your foot:**							
1a)Today my foot feels cold							
1b)Today my foot feels warm							
1c) Today my foot felt sensitive							
1d) Today my foot did not feel sensitive							
1e) There is no change in my foot at all →							
1f) Today I am unable to feel as much in my foot ↓							
1g) Today I can feel my foot more ↑							

**2. About your treatments:** (Please tick any that apply to you)	Sun	Mon	Tue	Wed	Thur	Fri	Sat
2a) Today the mobilization and tactile stimulation (MTS) was uncomfortable							
2b) The discomfort lasted for a long time e.g. several hours							
2c) The discomfort did not last long e.g. less than one hour							
2d) Today the mobilization and tactile stimulation (MTS) was **NOT** uncomfortable							
2e) Today the task specific walking training was uncomfortable							
2d) Today the task specific walking training was **NOT** uncomfortable							
2e) Today the outcome measurements were uncomfortable							
2f) Today the outcome measurements were **NOT** uncomfortable							
Additional comments:							

Supplementary information 3: Task-specific gait (walking) training (TSGT) schedule for recording treatments delivered

NB Standard exercises for TSGT will be delivered (20 sessions of 30 minutes), however, they will be individualised for each participant according to need. It is essential that the research therapist liaises with the clinician responsible for routine therapy treatment for the participant, to ensure the participant does not become fatigued by the extra therapy.

Description	Task	Tick (If done)	Details
**Warm Up**	Stepping in sitting		
	Reaching/Rolling gymball forwards in sitting		
	Pelvic tilt – ant/post/lateral		
	Hip flexion/inner range quads		
	Ankle circles/dorsiflexion/plantar flexion/heel/toe		
	Stretching of the trunk, thigh, and calf muscles		
	Weight transfer in standing		
	Marching on-the-spot		
Other			
**Lying Exercises**	Lifting leg on/off block		
**Sitting Exercises**	Postural re-education		
	Sitting at a table and reaching in different directions for objects located beyond arm's length to promote loading of the affected leg and activation of affected leg muscles		
	Lifting leg on/off block		
	Heel lifts in sitting to strengthen the affected plantar muscles		
	Rolling ball with foot/kicking ball		
	Wobble board/balance exercises		
	Wobble cushion in sitting		
	Heel lift in sitting		
	Theraband/strengthening		
	Exercise bike		
Other:			
**Sit to stand to sit**	Sit to stand from various chair heights to strengthen the affected leg extensor muscles and practice this task		
	One leg stand/foot not affected by stroke in front/on step		
Other:			
**Standing**	Weight shift left/right		
	Standing and reaching		
	Standing with the base of support constrained, with feet in parallel and tandem conditions reaching for objects, including down to the floor, to improve standing balance.		

	Heel lifts in standing to strengthen the affected plantar flexor muscles		
	Reciprocal leg flexion and extension in standing to strengthen leg muscles		
	'standing balance'/balance control		
	Step standing		
	Kicking ball with either foot		
	Balance beam - walking forwards/sidewards/backwards between two parallel lines, 20 cm apart, progressing to using one line, to using a balanced beam, and finally to lateral stepping on the floor, feet crossing over in front or in back, and then alternating		
	Bilateral squats		
	Stand on one leg		
	Squat on one leg		
	Stand on the paretic leg, then perform plantarflexion		
Other:			
**Stepping**	Stepping forwards/backwards/sideways onto blocks of various heights to strengthening affected leg muscles.		
	Stair climbing and descending exercise progressing from taking one step at a time to taking alternating steps, from using then not using the handrail, and to achieving a greater number of flights		
	Step ups progressed by increasing the height of the step, reducing arm support		
	Step on block with the paretic limb		
	Step on block with the non-paretic limb		
	Step sideways on a block		
Other:			
**Gait**	Standing up from a chair, walking and short distance, and returning to the chair to promote a smooth transition		
	Walking on a treadmill		
	Walking over various surfaces and obstacles		
	Walking in different directions		
	Tandem walking		
	Walking over slopes and stairs providing the opportunity for walking of practice under various- conditions		
	Inside and outside walking		
	Sudden stops and turns during walking		
	Walking on different surfaces (carpet, foam)		
	Standing on foam, balance disc, or wobble board		
	Walking through an obstacle course		
	Speed walking		
	Walk on foot prints		
	Walk between lines		
	Walking and picking up various objects from the ground		
	Gait re-education with or without aids (please specify)		
Other:			
**Functional Tasks**	Bed mobility		
	Turning whilst walking		

	Obstacle course e.g. stepping onto/along/down from an aerobics step, walking over a mat, or a ramp, and returning, progressing by increasing the heights and number of obstacles, and from completing the course walking forwards to walking backwards.		
	Walk and carry - continuous walking carrying a grocery bag, progressing to carrying a bag in each hand, to increasing the weight of the bag, to carrying a laundry basket, and to stopping on command.		
	Stairs		
	Kitchen work		
	Personal care		
	Leisure/hobbies		
Other:			
**Strengthening**	Progressive-resistive exercise program for paretic hip flexors/extensors and ankle dorsiflexors/plantar flexors.		
	Progressive-resistive exercise program for paretic knee flexors and extensors		
	Partial squats: progressed by increasing movement magnitude		
	Toe rises: progressed from bilateral to unilateral rises on either side		
	Leg press		
Other:			
**Stretching**	Calf stretch - Standing with affected knee straight and extended arm/s resting on the wall: keeping the body straight, pivot the body forward at the ankles keeping heel on the floor until you feel the calf muscle is stretched. Hold for 2 minutes, relax, repeat 10 times.		
	Hamstring stretch in sitting		
Other:			
**Endurance/ Aerobic**	Bike (if available)		
	Treadmill (if available)		
	Brisk walking		
	Raising and lowering a 1.4-kg, 55-cm exercise ball (care taken of the shoulder)		
	Shuttle walking		
	Standing chest press		
Other:			

Progression

For specific strengthening - progress by increasing number of repetitions (e.g. from 2 sets of 10 to 3 sets of 10), according to the level of ability, and progress as they improve, also progress by reducing arm support. Other treatment progressions include increasing resistance e.g. in relation to gravity, increasing the range of movement or distance over which body weight is transported, changing weight of external objects, altering the height of seat/step and walking longer distances.

Supplementary information 4: Semmes Weinstein monofilaments (SWMs) protocol for testing

The Semmes Weinstein monofilament (SWM) testing allows for the assessment of light touch sensation. The calibrated 20-piece SWM kit (Patterson Medical) should be used.
Four points of the plantar surface of the foot should be tested:

1. Under the heel, in the midline of the foot, 1 cm forwards of the back of the heel.
2. Under the pad of the hallux
3. Under the 1st Metatarsal joint
4. Under the 5th metatarsal head

Commence by explaining the testing procedure to the participant and letting them feel the 2.83 SWM, on the hand (thenar eminence), of the side which is **less affected** by their stroke.

Then proceed to test each point in turn using the following procedure:
When applying the stimulation, you should take 1.5 seconds to apply the SWM, leave it in touch with the skin for 1.5 seconds and then remove for 1.5 seconds (one touch – do not repeat). The instruction to the participant is 'tell me when you can feel something'. NB Filaments must be applied at a 90-degree angle to the skin which is being tested. [1]

Touch -test sensory evaluator

	SWM Code	Target Force (grams)	Threshold
20	1.65	0.008	Normal plantar threshold sensation
19	2.36	0.02	
18	2.44	0.04	
17	2.83	0.07	
16	3.22	0.16	
15	3.61	0.4	
14	3.84	0.6	Diminished light touch
13	4.08	1	
12	4.17	1.4	
11	4.31	2	
10	4.56	4	Diminished protective sensation
9	4.74	6	
8	4.93	8	
7	5.07	10	Loss of protective sensation
6	5.18	15	
5	5.46	26	
4	5.88	60	
3	6.10	100	
2	6.45	180	
1	6.65	300	Deep pressure sensation only

[1] Adapted from 2000 North Coast Medical Inc. Morgan Hill

Commencing at SWM 2.83 (which is in the middle of the normal threshold for plantar threshold normative touch-pressure data). Please follow the flow chart below:

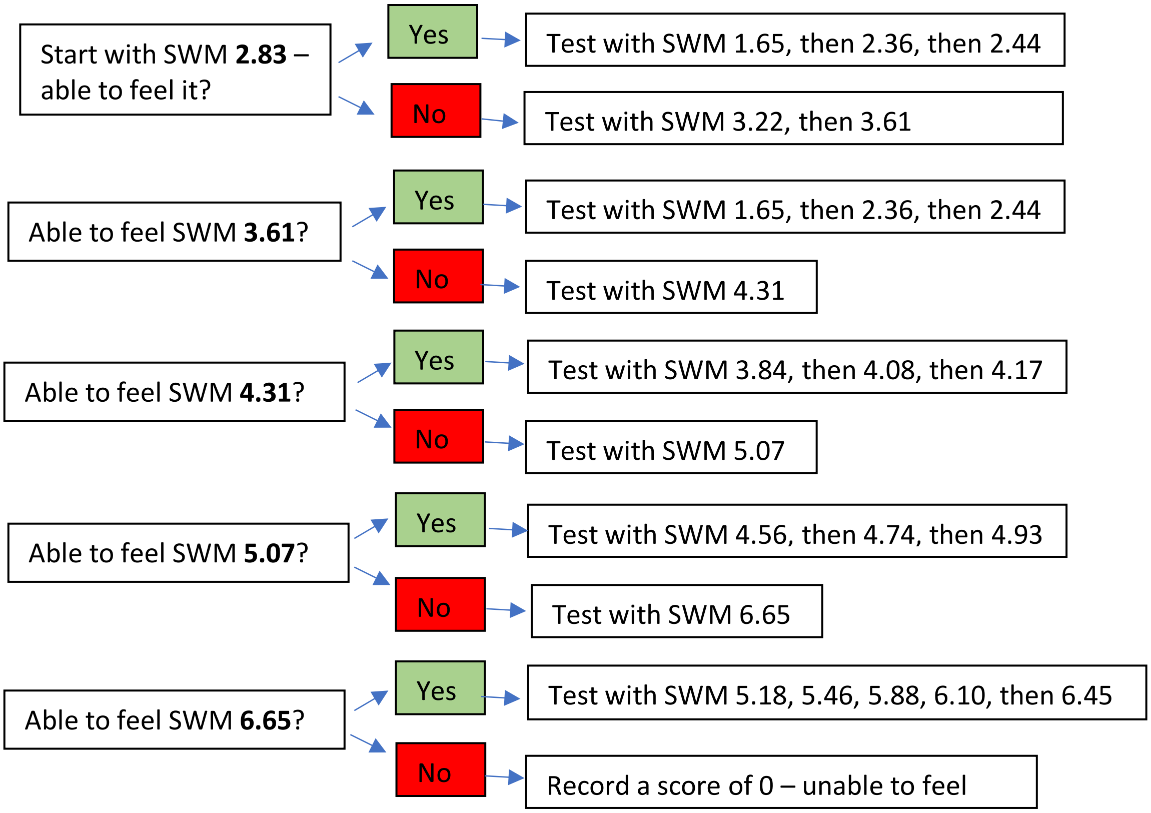

The decision to apply a thinner or thicker diameter SWM is determined by the subject's positive or negative detection of the stimulus. A positive detection is observed when subjects indicate verbally that they feel the stimulus within 1 second of the stimulus being removed, by saying "yes." If subjects do not indicate they feel the stimulus or have a delayed response (saying "yes" greater than 1 second following removal of the stimulus), it should be considered a negative detection. A positive detection should lead to a thinner diameter being tested. A negative detection (i.e., no verbal response) should lead to a thicker diameter SWM being tested. Time between stimuli should be varied so the subject does not preempt the application pattern. The SWM perceived most consistently should be recorded as the sensation threshold. Thicker diameter SWMs involve a greater force and are associated with decreased light touch sensitivity.

NB For baseline and end of intervention both sides should be assessed with the SWMs, however, for all other assessments just the affected foot should be assessed.

Supplementary information 5a: Focus group schedule for the mobilization and tactile stimulation (MTS) focus groups

Title: Sensory stimulation of the foot and ankle early post stroke: a feasibility study (MoTaStim-Foot)

Date:

Nature of Group: Mobilization and tactile stimulation (MTS) group

Name of Facilitator: Alison Aries

Name of Note taker:

Introduction to the process

Thank the participants for agreeing to attend a focus group discussion regarding their participation in the study mobilization and sensory stimulation of the foot and ankle post stroke: a feasibility study (MoTaStim-Foot). Provide the participant with a copy of the consent form which has previously been completed. Answer any questions that may arise as comprehensively as possible. Emphasise to the participant that:

1. The focus group interview will take no longer than one and a half hours.
2. To accurately capture what is being said the interview will be audiotaped
3. All information that is collected about the participant during the course of the study will be kept strictly confidential. Everyone needs to respect this please.
4. Any participants will remain anonymous in any dissemination work undertaken external or internal to the University.
5. In addition to consenting to be interviewed, the participant will also have given consent for direct quotations from the interview to be used in the write up of the evaluation. Please note that should you choose to withdraw at any point in time any information already collected will be used.
6. Any quotations that are used will be completely anonymous.
7. The information provided by the participant will be used to inform a future study looking at mobilization and tactile stimulation to the foot post stroke and in dissemination activities (conference presentations/ paper etc).

Ensure that the above points have been fully considered by the participant. Ask if they have any questions and then

Introduce the team:

When verbal consent on the day has been obtained, ask the participants if it is OK to turn on the tape recorder and conduct the interview. Remind people that there is no right or wrong answer and they should just give their honest opinion. Switch on the audiotape.

Focus Group Schedule

The following topic areas will be explored and similar questions to those below will be asked:

A. **About the interventions –** Remind participants that they had two different types of intervention at each treatment session – mobilization and tactile stimulation and task-specific walking training.

1.0 Mobilization and tactile stimulation (MTS) - I want to find out more about how the MTS felt

1.0 Can you tell me how you found the mobilization and tactile stimulation (MTS)?

More specifically:

I want to find out more about how the treatment felt

1.1 If you were describing the MTS treatment to someone else – from your experience what would you say?
1.2 Was it comfortable or uncomfortable? – What did you do if it became uncomfortable?
1.3 How long did any discomfort last?
1.4 Have you any other comments to make about the treatment?

2.0 Task-specific gait training: I want to find out more about how the task-specific walking training felt.

2.1 If you were describing the task-specific walking training treatment to someone else – from your experience, what would you say?
2.2 Was it comfortable or uncomfortable? – What did you do if it became uncomfortable?
2.3 How long did any discomfort last?
2.4 Have you any other comments to make about the treatment?

3.0 How was it for you when you had your treatments?

3.1 Treatment took place regularly – can you tell us how it felt to have people coming to you that regularly?
3.2 How easy was it to access your treatment?...or did you have your treatment at home?
3.3 Did you feel comfortable with the situation?
3.4 How did you feel when the treatments finished?

B. **About the outcome measurements:**

4.0 Can you say a little about the outcome measures?

4.1 Were the outcome measures uncomfortable or difficult?
4.2 Was there anything in particular about a specific outcome measure and why?

When you went for the outcome measures, can you say what was it like?
4.3 Was it easy to find?
4.4 Did you have to travel far?
4.5 Did you feel comfortable?

C. About the daily diaries:
5.0 Can you say a little about using the daily diaries?
5.1 How did you find filling in the daily diaries?
5.2 Can you say how easy or difficult you found them to complete?
5.3 Did you develop any particular patterns for filling them in?
5.4 Did you personally get anything from filling in the diaries?
5.5 Can you make any suggestions for changing the diaries in any way?

D. About any changes the participants may have perceived:
6.0 Have you noticed any changes?
6.1 Have you noticed any differences in your foot at all?
6.2 Can you say if your ability to walk / function has changed in any way?
6.3 Can you say if your confidence whilst walking changed at all?
6.4 Has the treatment made any difference to your lives?
6.5 What impact has it had on you....and your family?
6.6 Have we helped you achieve your goals for the future?

Please can you describe your experience on the trial in one word or one sentence?

Is there anything else you would like to tell me about your experiences of MTS and task-specific gait training?

Thank you for your time today.

Debriefing session - Check if there is anything else which should be added.

Supplementary information 5b: Focus group schedule for the textured insole (TI) groups

Title: Sensory stimulation of the foot and ankle early post stroke: a feasibility study (MoTaStim-Foot)

Date:

Nature of Group: Textured insoles group

Name of Facilitator: Alison Aries

Name of Note taker:

Introduction to the process

Thank the participants for agreeing to attend a focus group discussion regarding their participation in the study mobilization and sensory stimulation of the foot and ankle post stroke: a feasibility study (MoTaStim-Foot). Provide the participant with a copy of the consent form which has previously been completed. Answer any questions that may arise as comprehensively as possible. Emphasise to the participant that:

1. The focus group interview will take no longer than one and a half hours.

2. To accurately capture what is being said the interview will be audiotaped.

3. All information that is collected about the participant during the course of the study will be kept strictly confidential. Everyone needs to respect this please.

4. Any participants will remain anonymous in any dissemination work undertaken external or internal to the University.

5. In addition to consenting to be interviewed, the participant will also have given consent for direct quotations from the interview to be used in the write up of the evaluation. Please note that should you choose to withdraw at any point in time any information already collected will be used.

6. Any quotations that are used will be completely anonymous.

7. The information provided by the participant will be used to inform a future study looking at mobilization and tactile stimulation to the foot post stroke and in dissemination activities (conference presentations/ paper etc).

Ensure that the above points have been fully considered by the participant. Ask if they have any questions.

Introduce the team:

When verbal consent on the day has been obtained, ask the participants if it is OK to turn on the tape recorder and conduct the interview. Remind people that there is no right or wrong answer and they should just give their honest opinion. Switch on the audiotape.

Focus Group Schedule

The following topic areas will be explored and similar questions to those below will be asked:

A. **About the interventions –** Remind participants that they had two different types of intervention at each treatment session – Wearing textured insoles and task specific walking training.

1.0 Textured insoles - I want to find out more about what it was like to wear the textured insoles

1.1 Can you tell me how the textured insoles felt to wear?

More specifically:

I want to find out more about how the treatment felt

1.2 If you were describing what it was like to wear textured insoles to someone else – from your experience what would you say?
1.3 Was it comfortable or uncomfortable? – What did you do if they became uncomfortable?
1.4 How long did any discomfort last?
1.5 What could you say about the time given for you wearing the textured insoles?
1.6 Was the time too long / too short or, just right?
1.7 How might you describe the ease of wearing the textured insoles?
1.8 Were they easy to put in your footwear?
1.9 Have you any other comments to make about wearing textured insoles?

2.0 Task-specific gait training: I want to find out more about how the task-specific walking training felt.

1.1 If you were describing the task-specific walking training treatment to someone else – from your experience what would you say?
1.2 Was it comfortable or uncomfortable? – What did you do if it became uncomfortable?
1.3 How long did any discomfort last?
1.4 Have you any other comments to make about the treatment?

3.0 How was it for you when you had your treatments?

3.2 Treatment took place regularly – can you tell us how it felt to have people coming to you that regularly?
3.3 How easy was it to access your treatment?....or did you have your treatment at home?
3.4 Did you feel comfortable with the situation?
3.5 How did you feel when the treatments finished?

B. About the outcome measurements:

4.0 Can you say a little about the outcome measures?

4.1 Were the outcome measures uncomfortable or difficult?

4.2 Was there anything in particular about a specific outcome measure and why?

When you went for the outcome measures, can you say what was it like?

4.3 Was it easy to find?

4.4 Did you have to travel far?

4.5 Did you feel comfortable?

C. About the daily diaries:

5.0 Can you say a little about using the daily diaries?

5.1 How did you find filling in the daily diaries?

5.2 Can you say how easy or difficult you found them to complete?

5.3 Did you develop any particular patterns for filling them in?

5.4 Did you personally get anything from filling in the diaries?

5.5 Can you make any suggestions for changing the diaries in any way?

D. About any changes the participants may have perceived:

6.0 Have you noticed any changes?

6.1 Have you noticed any differences in your foot at all?

6.2 Can you say if your ability to walk / function has changed in any way?

6.3 Can you say if your confidence whilst walking changed at all?

6.4 Has the treatment made any difference to your lives?

6.5 What impact has it had on you?....and your family?

6.6 Have we helped you achieve your goals for the future?

Please can you describe your experience on the trial in one word or one sentence?

Is there anything else you would like to tell me about your experiences of TIs and task-specific gait training?

Thank you for your time today.

Debriefing session - Check if there is anything else that should be added.
